# Maternal red blood cell folate metabolites were dynamically associated with neonatal amino acids and acylcarnitines in heel blood: a prospective cohort study

**DOI:** 10.1186/s12986-025-01064-2

**Published:** 2026-01-07

**Authors:** Ruihua Yang, Wei Zheng, Yixuan Lu, Yujie Zhang, Lirui Zhang, Xin Yan, Tengda Chen, Ziyu Wang, Xincong Shi, Yuanyuan Kong, Guanghui Li

**Affiliations:** 1 Department of Nutrition, Endocrinology, and Metabolism, Beijing Obstetrics and Gynecology Hospital, Capital Medical University, Beijing, China; 2 Beijing Maternal and Child Health Care Hospital, Beijing, China; 3 Department of Newborn Screening Center, Beijing Obstetrics and Gynecology Hospital, Capital Medical University, Beijing, China

**Keywords:** Folate, Neonatal metabolism, Amino acids, Acylcarnitines

## Abstract

**Background:**

Animal studies indicate maternal folic acid intake alters offspring amino acids (AAs) and fatty acid profiles, affecting their long-term health. However, human data on specific folate metabolites remain limited. We explored their dynamic associations with neonatal AAs and acylcarnitines (ACs).

**Methods:**

We conducted a prospective cohort study, involving 4130 singleton pregnant women and their neonates. Maternal total folate and related metabolites in red blood cell (RBC), including 5‐methyltetrahydrofolate (5‐MTHF), tetrahydrofolate (THF), 5‐formyltetrahydrofolate (5‐CHO‐THF), and unmetabolized folic acid (UMFA), were measured in 6–17 (T1), 20–26 (T2), and 32–36 (T3) gestational weeks. Neonatal metabolites, including 11 AAs and 31 ACs, were routinely tested 72 h postpartum using heel blood samples. Linear regression and pathway analysis were used to evaluate associations between maternal folate metabolites with neonatal metabolic pathways.

**Results:**

5-MTHF and total folate levels significantly increased from T1 to T2 (*p* < 0.001), and stabilized from T2 to T3 (*p* > 0.05). THF and 5-CHO-THF levels showed a continuous upward trend (*p* < 0.001). Conversely, UMFA declined throughout pregnancy (*p* < 0.001). Total folate and 5-MTHF showed no significant correlations with neonatal metabolism. THF were associated with multiple neonatal metabolites, and the associations peaked in T1 and declined in T2 and T3. THF in T1 positively correlated with phenylalanine and tyrosine, involved in the phenylalanine and tyrosine metabolism. And it was positively associated with 7 ACs and negatively with 9 ACs, involved in the fatty acids β-oxidation. THF in T2 or T3 positively related to arginine, and negatively with citrulline, glycine, and ornithine, involved in the urea cycle and arginine and proline metabolism. 5-CHO-THF displayed similar and weaker impacts, correlated with fatty acids β-oxidation only in T1. UMFA exhibited different and weaker influence, related to the urea cycle, arginine and proline metabolism only in T1.

**Conclusions:**

Although maternal total folate and 5-MTHF levels showed no significant association with neonatal metabolites, maternal certain folate metabolites, especially THF, exhibited trimester-specific associations with neonatal metabolic pathways, warranting clinical attention.

**Supplementary Information:**

The online version contains supplementary material available at 10.1186/s12986-025-01064-2.

## Background

Folate, vitamin B9, is crucial for nucleotide synthesis, amino acid metabolism, and methylation reactions, playing a pivotal role in embryonic development [1]. Its synthetic form, folic acid (FA), exerts physiological effects after being converted into its active derivatives. FA is first activated to tetrahydrofolate (THF), then sequentially converted into 5-formyltetrahydrofolate (5-CHO-THF), 5,10-methylenetetrahydrofolate (5,10-CH2-THF), and ultimately 5-methyltetrahydrofolate (5-MTHF), the predominant circulating folate form. 5-MTHF can be reconverted to THF to sustain metabolic cycling [2]. Among these, 5,10-CH2-THF is essential for DNA synthesis, aiding in the conversion of deoxyuridine monophosphate (dUMP) to deoxythymidine monophosphate (dTMP). 5-MTHF participates in the one-carbon metabolism through the methionine cycle. THF serves as the central hub in folate metabolism, functioning as both the precursor for all active folate forms and the core of the metabolic cycle [2, 3]. Unmetabolized folic acid (UMFA) can accumulate under excessive FA intake, although current evidence regarding its potential toxicity in humans remains inconclusive [3]. Notably, 5-CHO-THF is recognized as a key storage form of folate within cells. It is the only folate vitamer that is chemically stable and does not serve as a direct cofactor. This stability allows it to act as a reservoir for one-carbon units, functioning as a buffer to regulate the cellular pool of active folate cofactors (e.g., 5,10-CH2-THF) [4, 5]. Consequently, quantifying 5-formyl-THF provides valuable insight into the reserve capacity and homeostasis of the folate-mediated one-carbon metabolism. Although 5-CHO-THF, along with THF and UMFA, is not the predominant circulating form, their measurements help construct a more comprehensive folate metabolic profile, enabling a better assessment of the body's folate status and metabolic dynamics. The folate metabolism is an intricate process, influenced by vitamin B12 levels, homocysteine (Hcy) levels, as well as polymorphisms in the methylenetetrahydrofolate reductase (MTHFR) gene and the methionine synthase reductase (MTRR) gene [3].

FA supplementation during periconception is widely recommended to prevent fetal neural tube defects (NTDs) [6]. Maternal FA supplement reduced the risk of small for gestational age (SGA) and low birth weight (LBW) births [7, 8]. However, concerns arise about its inappropriate intake, and the adverse consequences stretch beyond the immediate effects on the mother, potentially influencing future generations through epigenetic reprogramming [9]. Current research indicates that maternal folate levels may exhibit a U-shaped association with offspring's diseases [10]. The absence of maternal FA supplementation before and during early pregnancy can elevate the risk of fetal congenital heart defect (CHD) [11], obesity [12], and abnormal neurodevelopmental outcomes in offspring [13]. Quantitative studies have confirmed that inadequate maternal total folate levels in red blood cell (RBC) before or during early pregnancy increase CHD risk in offspring [14]. However, high prenatal FA exposure may also increased the risk of large for gestational age (LGA) infants [15], and predispose offspring to cancer [8, 9, 16], neurodevelopmental impairment [9], cardiac dysfunction [17], and asthma [8, 18].

Animal studies have revealed that maternal FA supplementation during pregnancy can lead to changes in the metabolic processes of offspring, which significantly affects their long-term metabolic health and neurodevelopment [19, 20]. These metabolic changes are characterized by alterations in amino acids (AAs) and fatty acids [19, 20]. Pups in FA supplement group had significantly lower serum homocysteine and higher folate levels than control pups, confirming the mechanism of placental transfer [19]. A reduced THF availability disrupts serine hydroxymethyltransferase (SHMT)-mediated interconversion of serine (Ser) and glycine (Gly). This metabolic disruption indirectly impairs the production of pyruvate, impacting branched-chain amino acids metabolism [19, 20]. The folate cycle can influence lipid metabolism through methylation reactions by providing methyl groups via 5-MTHF to regenerate methionine, thereby producing S-adenosylmethionine (SAM), the key methyl donor. Phosphatidylethanolamine N-methyltransferase (PEMT) is the key enzyme that catalyzes the conversion of phosphatidylethanolamine (PE) to phosphatidylcholine (PC), a process that depends on SAM. S-adenosylhomocysteine (SAH), the demethylated product of SAM, acts as an inhibitor of PEMT. Elevated SAH concentrations may therefore reduce PC synthesis efficiency by suppressing PEMT activity [21, 22]. Furthermore, due to the upregulation of PEMT by estrogen, females may inherently exhibit higher PEMT activity, thereby enhancing PC synthesis [21, 22]. The PEMT-mediated synthesis of PC preferentially incorporates docosahexaenoic acid (DHA), leading to its accumulation. As an end-product of n-3 polyunsaturated fatty acid (PUFA) metabolism, DHA indirectly inhibits Δ5-desaturase (D5D) activity, a key enzyme in the n-6 pathway, primarily by depleting shared cofactorsand downregulating D5D expression, thereby reducing its catalytic efficiency toward n-6 substrates, such as dihomo-γ-linolenic acid (DGLA). Concurrently, the remodeling of membrane phospholipids (e.g., increased PC and lysophosphatidylcholine) driven by PEMT activity alters the lipid microenvironment, further limiting the accessibility of arachidonic acid (AA) precursors and promoting competitive displacement of AA from membrane sites. These combined mechanisms result in a significant reduction in AA availability [19, 20]. However, human studies regarding maternal folate levels on offspring metabolism remain limited.

Previous human studies primarily focus on exploring the effects of maternal FA supplementation on the disease risks in offspring [6–9, 11–13, 15–18]. However, FA intake doses cannot objectively reflect the true folate levels in the human body due to the multifactorial regulation of folate metabolism. Moreover, current human research overlooks the distinct roles of individual folate metabolites and lacks dynamic monitoring, leaving their trimester-specific effects on offspring metabolism unclear. Researches have showed that RBC folate parallels liver concentrations (accounting for ∼50% of total body folate) and is thus considered to reflect tissue folate stores [23], and it can reliably reflect the sustained folate levels [24]. Several methods are available to measure folate levels. Radioassays are fast and widely used for detecting plasma folate, but only provide total folate concentrations and can be highly sensitive to assay conditions. Microbiological assays detect total folate relatively easily but require multiple steps to measure individual metabolites. LC–MS/MS methods offer high specificity, reliable internal standards, and simultaneous quantification of multiple folate metabolites [2]. Additionally, in our region, the levels of 11 AAs and 33 acylcarnitines (ACs) in neonates were routinely tested 72 h postpartum using heel blood samples, with the latter being essential mitochondrial transporters of fatty acid β-oxidation, indirectly reflecting fatty acid metabolism.

**Fig. 1:**
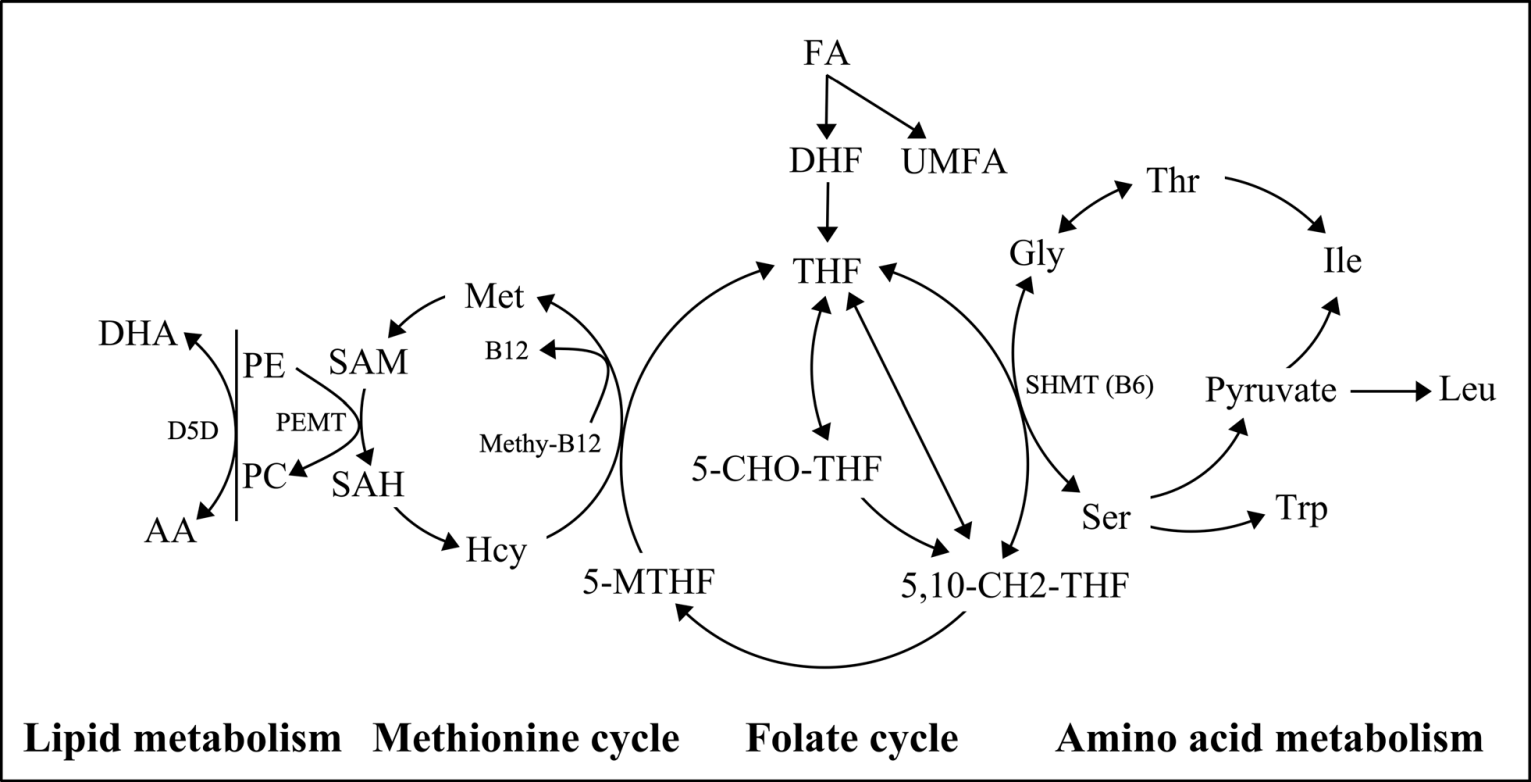
Metabolic pathways of folate cycle, methionine cycle, amino acid metabolism and lipid metabolism. Abbreviations: DHA docosahexaenoic acid, AA arachidonic acid, D5D Δ5-desaturase; PE phosphatidylethanolamine, PC phosphatidylcholine, PEMT phosphatidylethanolamine N-methyltransferase, Met methionine, Hcy homocysteine, SAM S-Adenosylmethionine, SAH S-Adenosylhomocysteine, FA folic acid, DHF dihydrofolate, UMFA unmetabolized folic acid, THF tetrahydrofolate, 5-CHO-THF 5-formyltetrahydrofolate, 5,10-CH2-THF 5,10-methylenetetrahydrofolate, 5-MTHF 5-methyltetrahydrofolate, SHMT serine hydroxymethyltransferase, Ser Serine, Gly Glycine, Thr Threonine, Ile Isoleucine, Leu Leucine, Trp Tryptophan

Therefore, this study explored the dynamic associations between maternal folate metabolites levels in RBC throughout pregnancy and neonatal AAs and ACs levels, and further analyzed their correlations with neonatal metabolic pathways. We aimed to identify trimester-specific effects of critical folate metabolites on offspring amino acid and fatty acid metabolism (Fig. 1).

## Methods

### Study design and participants

This study was a prospective cohort study conducted at Beijing Obstetrics and Gynecology Hospital, from April 2022 to June 2024 (ChiCTR2400090880, registered at https://www.chictr.org.cn/index.html). This cohort recruited singleton pregnant women between 6 and 13 gestational weeks and followed them until delivery. Participants were required to undergo folate metabolites testing in the early pregnancy, middle pregnancy, and late pregnancy. Additionally, their neonates underwent peripheral blood AAs and ACs screening.

The inclusion criteria encompassed singleton pregnant women, 18–44 years old, 6–13 gestational weeks, and engaging in regular prenatal health check‐ups at the research centre. Women with chronic medical conditions (hypertension, diabetes, dyslipidemia, thyroid disease, and moderate or severe anemia), gastrointestinal diseases, infectious diseases (hepatitis and tuberculosis), or mental illness were not included. Women who lacked baseline information, maternal folate metabolites testing data during pregnancy or neonatal metabolite results were excluded from the study.

**Fig. 2:**
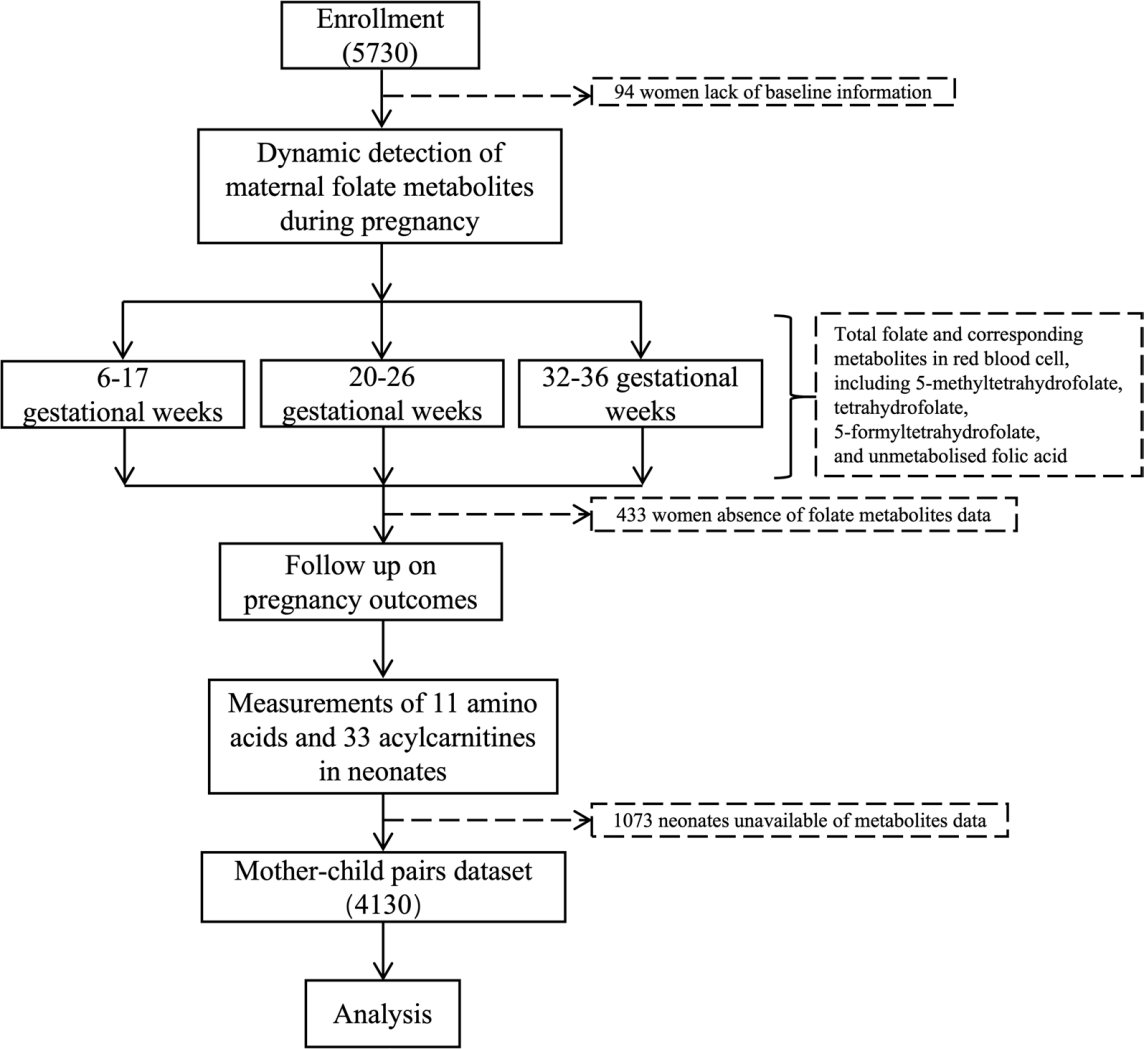
A flow diagram of participants

The screening was conducted on the original group of 5730 women, excluding 94 women lack of baseline information, 433 women absence of folate metabolites data, 1073 neonates unavailable for metabolites data, resulting in a final data set of 4130 mother–child pairs (Fig. 2). This research adhered to the ethical guidelines in the Helsinki Declaration and received approval from the Ethics Committee of the Beijing Obstetrics and Gynecology Hospital (2022‐KY‐021‐01 and 2023-KY-050-01). Prior written consent was obtained from all participants or their legal guardians.

### Collection of baseline and FA supplement information

Detailed participant characteristics were collected by qualified researchers upon enrollment, including age, height, pre-pregnancy weight, gestational weeks at enrollment, ethnicity, educational levels, occupation, economic state, smoking and drinking habits, gravidity and parity, history of pregnancy and childbirth history, and family history of hypertensive disorders and diabetes. The pre-pregnancy body mass index (BMI) was calculated by dividing the pre-pregnancy weight (kg) by the square of the height (m). Furthermore, the brand, daily dosage and commencement time of FA intake from supplements before and during pregnancy were surveyed by questionnaires at enrollment. The daily dosage of FA intake is calculated by multiplying the content per tablet stated in the product instructions by the number of tablets taken per day. Dietary folate intake was not assessed.

### Maternal folate metabolites and folate genotypes measurements

Venous whole blood samples were collected by nurses using ethylenediaminetetraacetic acid (EDTA) anticoagulant tubes after an overnight fast during 6–17 (T1), 20–26 (T2), and 32–36 (T3) gestational weeks. A minimum volume of 500–600 μL was drawn per sample. Each tube was labeled with a unique barcode and immediately stored at − 20 °C. Samples were tested within one week of collection. This study included 4130 women who had folate metabolites levels assessed during at least one period. Specifically, 3222 blood samples were collected in T1, 3287 in T2, and 3111 in T3.

Total folate and corresponding metabolites levels in the whole blood hemolysate solution, including 5-CHO-THF, THF, 5-MTHF and UMFA, were measured using LC–MS/MS with an ultra performance liquid chromatography (UPLC)‐triple quadrupole mass spectrometer. Our LC–MS/MS methodology, aligned with the core steps of previous studies [2, 25–27], implemented a multi-faceted strategy to ensure the measured value reliably reflects the original sample content. Key components included standards (FA, THF, and 5-CHO-THF from Sigma-Aldrich; and 5-MTHF from Toronto Research Chemicals, and isotope-labeled internal standards (FA−^13^C5 from Sigma-Aldrich and 5-MTHF-^13^C5 from Cambridge Isotope Laboratories) for accurate quantification. Calibration standards were prepared at different concentration ranges: 10–1000 ng/mL for 5-MTHF in whole blood and 0.5–50 ng/mL for THF, 5-CHO-THF, and UMFA in whole blood. Both calibration standards and test samples were analyzed simultaneously during each run, and samples showing significant deviations from the limit of quantification were reanalyzed to ensure data reliability.

Samples were thawed at room temperature under light protection. After vortexing, 100 µL aliquots were mixed with 10 µL internal standard solution (200 ng/mL in 20% methanol–water) and 700 µL dilution buffer (containing 1% sodium ascorbate, 0.5% mercaptoethanol, and 0.05% 5 M NaOH), followed by heating at 90 °C for 15 min, the use of sodium ascorbate and mercaptoethanol effectively protected specific folate metabolites from oxidation, largely stabilizing the folate forms. The rapid 4 °C centrifugation (15,000 rpm, 10 min,) was employed to avoid thermal degradation, and then 700 µL supernatant was incubated with 30 µL charcoal-stripped rat serum (a source of conjugase enzymes) at 37 °C for 5 h to hydrolyze polyglutamates to monoglutamates to ensure analytical fidelity. Cleanup was performed using Oasis MAX µElution plates (2 mg sorbent) preconditioned with methanol and 0.2% ammonia. Samples were loaded, washed with water, and eluted with 2% formic acid in methanol (containing 1% dilution buffer). Eluates were dried under nitrogen at 50 °C, reconstituted in 100 µL water, and centrifuged before LC–MS/MS analysis (5 µL injection). All procedures were conducted under red light to prevent photodegradation. We ensured rapid processing and control of the pH and incubation time for each buffer step to possibly minimize analyte exposure to extreme pH conditions for reducing pH-dependent interconversion of different folate metabolites [28].

Analysis was performed on the Waters Masslynx 4.1 system connected to a Waters TQS system with electrospray ionization in positive multiple reaction monitoring (MRM) mode. Key parameters: desolvation gas 1000 L/h, cone gas 150 L/h, desolvation temperature 600 °C, capillary voltage 3.0 kV. Separation used an Acquity UPLC HSS T3 column (50 × 2.1 mm, 1.8 µm) with 0.1% formic acid in water (A) and methanol–acetonitrile (1:1, v/v; B) as mobile phases (0.4 mL/min, 4 min total run time). Gradient: 0 min, 95% A, 5% B; 1.50 min, 85% A, 15% B; 2.00 min, 20% A, 80% B; 2.50 min, 20% A, 80% B; 2.51 min, 95% A, 5% B; 3.00 min, 95% A, 5% B, quantified accurately using internal standard method to correct for recovery losses and matrix effects. Quantitation was performed using the following transitions: FA (m/z 442.2 → 295.2), FA-C₅ (m/z 447.1 → 295.2), THF (m/z 446.2 → 299.2), 5-CHO-THF (m/z 474.2 → 327.2), 5-MTHF (m/z 460.2 → 313.3), and 5-MTHF-^13^C₅ (m/z 465.2 → 313.3), with isotope dilution calibration.

Notably, accurately quantifying 5-CHO-THF is challenged by MeFox, an oxidation degradation product of 5-MTHF formed during sample storage or processing. MeFox and 5-CHO-THF are isobaric compounds and as such form the same mass to charge parent to product ion pairs during ionization, making coelution and misidentification likely [27]. To overcome this challenge, chromatographic conditions were optimized to achieve baseline separation of 5-CHO-THF and MeFox. Mass spectrometric separation was achieved by monitoring unique fragment ions (5-CHO-THF: m/z 474.2 → 327.2) because 5-CHO-THF and MeFox generate distinct characteristic product ions. The coefficients of variation for these measurements were as follows: 2.5–8.6% for 5‐MTHF, 3.8–9.3% for THF, 3.4–7.0% for 5‐CHO‐THF, and 2.0–5.4% for UMFA.

The folate metabolites in RBC were calculated by dividing the folate concentration in the whole blood hemolysate solution by the haematocrit value [29]. Furthermore, RBC total folate refers to the sum of UMFA, 5‐MTHF, THF, and 5‐CHO‐THF in RBC, as these are stable components during folate metabolism [2]. The MTHFR and MTRR polymorphisms were genotyped using polymerase chain reaction (PCR)‐based KBiosciences Competitive Allele‐Specific PCR genotyping system (KASP) assays (Hoddesdon, UK).

### Collection of pregnancy outcomes

Professional medical personnel tracked participants' pregnancy outcomes from the medical system of the hospital, including hypertensive disorders in pregnancy (HDP), gestational diabetes mellitus (GDM), gestational week at delivery, delivery mode, neonatal gender and birth weight. HDP was characterized by hypertension with or without proteinuria after 20 gestational weeks, encompassing gestational hypertension, preeclampsia, and eclampsia [30]. GDM diagnosis was based on 75 g oral glucose tolerance test (OGTT) results at 24–28 gestational weeks [31]. Neonates were categorized by weight as follows: large for gestational age (LGA, > 90th percentile of the same gender at the same gestational age), SGA (< 10th percentile of the same gender at the same gestational age) [32], macrosomia (> 4000 g), and LBW (< 2500 g) [33]. Preterm birth was defined as birth between 28 and 37 gestational weeks [34].

### Neonatal metabolites measurements

4130 newborns had complete data on 11 AAs and 31 ACs. Neonatal heel blood samples were collected 72 h postpartum as dried blood spots and stored at 4 °C. MS/MS was used to analyze 42 metabolites, comprising 11 AAs and 33 ACs extracted with methanol containing stable isotope-labeled standards based on Chinese expert consensus [35]. The supernatant was quantified via electrospray ionization using a Waters Xevo TQD Mass Spectrometer (Milford, MA) under settings: capillary, 3.20 kV; cone voltage and gas flow rate, 50 V, 50 L/h; desolvation temperature and gas flow rate, 350 °C, 800 L/h. The reference range of neonatal AAs and ACs of the study participant were listed in Table S3. The validity of measuring neonatal metabolites using dried blood spots were demonstrated in previous studies [36, 37].

### Statistical analysis

The characteristics of participants were presented as mean ± SD for continuous variables and n (%) for categorical variables. Maternal folate metabolites levels and neonatal metabolites levels were represented as median (interquartile range) and visualized through a line graph and a bar chart, respectively. The complete dataset was used for analysis. Paired t-tests with Bonferroni correction were used to conduct self-comparison of maternal folate metabolites from T1 to T2, and from T2 to T3, a corrected significance threshold of *p *< 0.025 (0.05/2) was applied to account for multiple comparisons. We conducted a linear regression analysis by IBM SPSS Statistics 27 software to examine the association between maternal folate metabolites levels at different stages of pregnancy and neonatal AAs and ACs, adjusted by maternal age, pre-pregnancy BMI, parity (primiparity or not), pregnancy complications (HDP and GDM), and neonatal gender. The maternal folate metabolites and neonatal metabolites levels both underwent log-transformation prior to the analysis. The Benjamini–Hochberg method was employed to control false positive errors in multiple testing, utilizing a false discovery rate (FDR) threshold of 0.05. Pathway analysis were conducted through the online MetaboAnalyst 6.0 tool (https://new.metaboanalyst.ca) to determine the potential impact of maternal folate metabolites levels on offspring's metabolic pathway. Pathway analysis evaluated the enrichment of neonatal metabolites relevant to maternal folate metabolites levels in biological pathways using hypergeometric test, and conducted topology analyses to calculate pathway impact values, referring to the Small Molecule Pathway Database (SMPDB). Notably, the differential neonatal metabolite names were first converted into corresponding Human Metabolome Database (HMDB) IDs to be inputted. GraphPad Prism 9 software was used to visualize. Additionally, we conducted a sex-specific analysis to investigate the association between the identified key maternal folate metabolites (THF) and neonatal metabolites.

## Results

### Maternal and neonatal clinical characteristics

This study encompassed a total of 4130 mother–child pairs. The mothers had an average age of 31.67 ± 3.70 years, with an average BMI of 22.00 ± 3.33 kg/m^2^, and 80.36% of the mothers were primiparas. Subsequently, 11.11% of the mothers developed HDP, and 22.45% were diagnosed with GDM. The average birth weight of neonates was 3289.61 ± 434.63 g. 10.19% of neonates were classified as LGA, 6.22% as SGA, 4.46% as macrosomia, 3.73% as LBW, and 4.89% as preterm birth (Table 1).

**Table 1:** Clinical characteristics of the participants

Maternal and neonatal characteristics	All (N = 4130)
Maternal characteristics	
Age (years)	31.67 ± 3.70
Height (cm)	162.83 ± 5.15
Pre-pregnancy weight (kg)	58.30 ± 9.15
Pre-pregnancy BMI (kg/m^2^)	22.00 ± 3.33
Primiparity	3319 (80.36%)
Gestational week of enrollment (weeks)	9.12 ± 2.52
Ethnicity	
Han	3843 (93.05%)
Others	287 (6.95%)
Education level	
Master or above	1148 (27.80%)
College or university	2891 (70.00%)
High school or below	91 (2.20%)
Occupation	
Technicians	1568 (37.97%)
Public official	1090 (26.39%)
Business or service	821 (19.88%)
Others	651 (15.76%)
Family income of average month	
30,000 CNY or above	2136 (51.72%)
10,000–30000 CNY	1907 (46.17%)
Below 10,000 CNY	31 (0.75%)
Refusal to inform	56 (1.36%)
Smoking during pregnancy	74 (1.79%)
Alcohol consumption during pregnancy	43 (1.04%)
History of pregnancy and childbirth history	
History of HDP	25 (0.61%)
History of GDM	73 (1.77%)
History of premature delivery	38 (0.92%)
Birth history of macrosomia	61 (1.48%)
Birth history of LBW infants	32 (0.77%)
History of cesarean section	132 (3.20%)
Family history of diseases	
History of hypertensive disorders	488 (11.82%)
History of diabetes mellitus	397 (9.61%)
Complications during this pregnancy	
HDP	459 (11.11%)
GDM	927 (22.45%)
FA intake from supplements before conception (μg/d)	
≥ 800	1022 (24.75%)
400–799	1352 (32.74%)
1–399	21 (0.50%)
0	1735 (42.01%)
FA intake from supplements after conception (μg/d)	
≥ 800	2033 (49.23%)
400–799	1890 (45.76%)
1–399	29 (0.70%)
0	178 (4.31%)
The MTHFR and MTRR gene polymorphisms (n = 3166)	
MTHFR 1298AA	2290 (72.33%)
MTHFR 1298AC	733 (23.15%)
MTHFR 1298CC	143 (4.52%)
MTHFR 677CC	639 (20.18%)
MTHFR 677CT	1509 (47.66%)
MTHFR 677TT	1018 (32.16%)
MTRR 66AA	1794 (56.67%)
MTRR 66AG	1200 (37.90%)
MTRR 66GG	172 (5.43%)
Neonatal characteristics	
Delivery gestational week (weeks)	38.84 ± 1.42
Cesarean section delivery	1843 (44.62%)
Male	2135 (51.69%)
Birth weight (g)	3289.61 ± 434.63
LGA	421 (10.19%)
SGA	257 (6.22%)
Macrosomia	184 (4.46%)
LBW	154 (3.73%)
Preterm birth	202 (4.89%)

Data are n (%), or mean ± SD. Abbreviations: BMI body mass index, CNY Chinese Yuan, HDP hypertensive disorders in pregnancy, GDM gestational diabetes mellitus, FA folic acid, MTHFR methylenetetrahydrofolate reductase, MTRR methionine synthase reductase, LGA large for gestational age, SGA small for gestational age, LBW low birth weight

42.52% women did not meet the recommended intake of 400 μg/d of FA before conception, but the percentage decreased to 5.01% during pregnancy. 24.75% women consumed ≥ 800 μg/d of FA before pregnancy, and the percentage increased to 49.23% during pregnancy. 42.01% women did not take FA supplements before pregnancy, but the percentage subsequently decreased to 4.31% during pregnancy. Additionally, 79.82% women exhibited the MTHFR 677CT/TT polymorphism, 27.67% had the MTHFR 1298AC/CC polymorphism, and 43.33% showed the MTRR 66AG/GG polymorphism, respectively (Table 1).

### An overview of maternal RBC folate metabolites and neonatal AAs and ACs levels

**Fig. 3:**
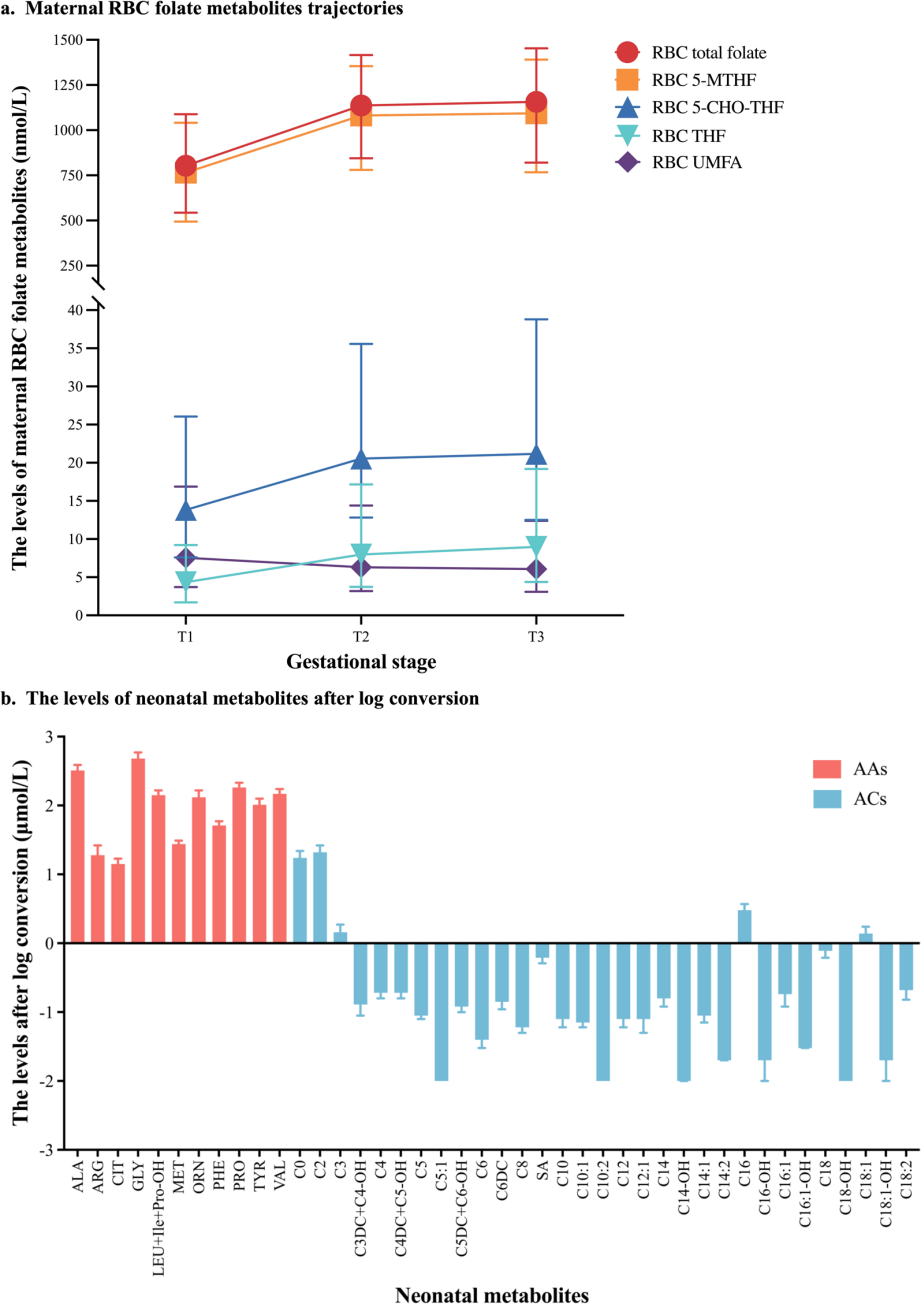
An overview of maternal folate metabolites (**a**) and neonatal metabolites (**b**). RBC: red blood cell, 5‐MTHF: 5‐methyltetrahydrofolate, THF: tetrahydrofolate, 5‐CHO‐THF: 5‐formyltetrahydrofolate, UMFA: unmetabolised folic acid, T1: 6–17 gestational weeks, T2: 20–26 gestational weeks, T3: 32–36 gestational weeks

Among the maternal RBC folate metabolites, 5-MTHF was the most predominant, whereas THF, 5-CHO-THF, UMFA constituted a small portion. 5-MTHF [in T1: 765.43 (493.75, 1041.34) nmol/L; in T2: 1081.42 (780.04, 1354.32) nmol/L; in T3: 1093.42 (766.62, 1390.62) nmol/L] and total folate levels [in T1: 803.71 (543.81, 1088.11) nmol/L; in T2: 1137.24 (845.04, 1415.63) nmol/L; in T3: 1156.54 (820.83, 1453.10) nmol/L] significantly increased from T1 to T2 (*p* < 0.001), and stabilized from T2 to T3 (*p* > 0.05). THF [in T1: 4.35 (1.70, 9.22) nmol/L; in T2: 7.97 (3.74, 17.17) nmol/L; in T3: 8.99 (4.37, 19.19) nmol/L], and 5-CHO-THF levels [in T1: 13.82 (7.59, 26.06) nmol/L; in T2: 20.55 (12.82, 35.57) nmol/L; in T3: 21.16 (12.51, 38.79) nmol/L] showed a continuous upward trend (*p* < 0.001). Conversely, UMFA [in T1: 7.55 (3.72, 16.88) nmol/L; in T2: 6.30 (3.19, 14.39) nmol/L; in T3: 6.07 (3.10, 12.40) nmol/L] declined throughout pregnancy (*p* < 0.001) (Fig. 3a and Table S1).

Among the AAs, the median levels of alanine (Ala) and Gly exhibited higher, whereas arginine (Arg) and citrulline (Cit) displayed relatively lower. The median levels of ACs were generally low, with only 5 ACs (C0, C2, C3, C16, C18:1) exceeding 1 μmol/L. Among them, C0 and C2 levels ranked at the top two positions (Fig. 3b and Table S2). All the AAs and ACs levels were generally within normal ranges (Table S2 and Table S3).

### Associations between maternal folate metabolites levels and neonatal metabolic pathways

**Fig. 4:**
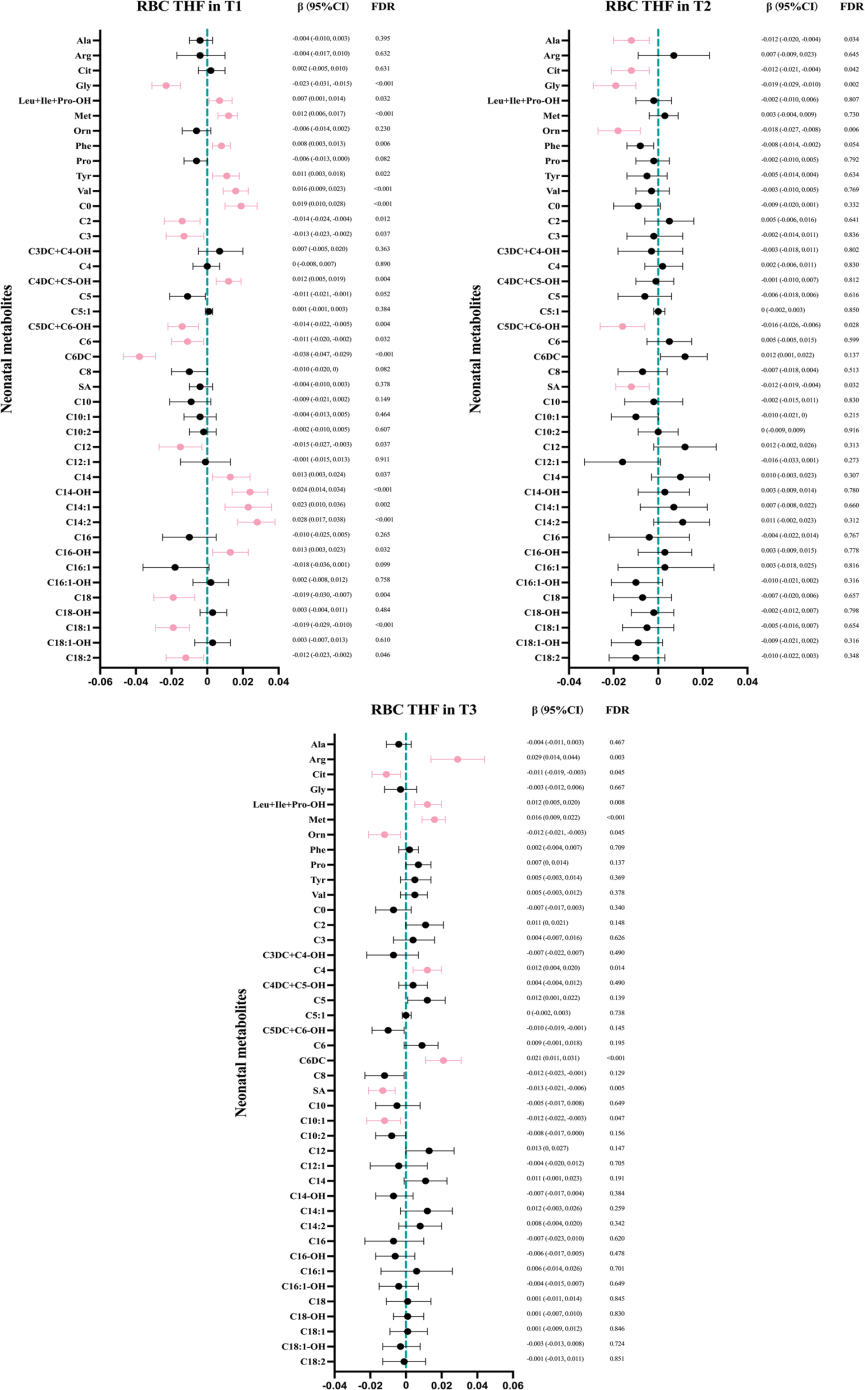
Associations between maternal RBC THF levels and neonatal metabolites. Maternal age, pre-pregnancy BMI, parity, HDP, GDM, and neonatal gender were adjusted in linear regression analysis. The FDR values were calculated to account for multiple comparisons using the Benjamini–Hochberg method. The red color indicates an FDR < 0.05

**Fig. 5:**
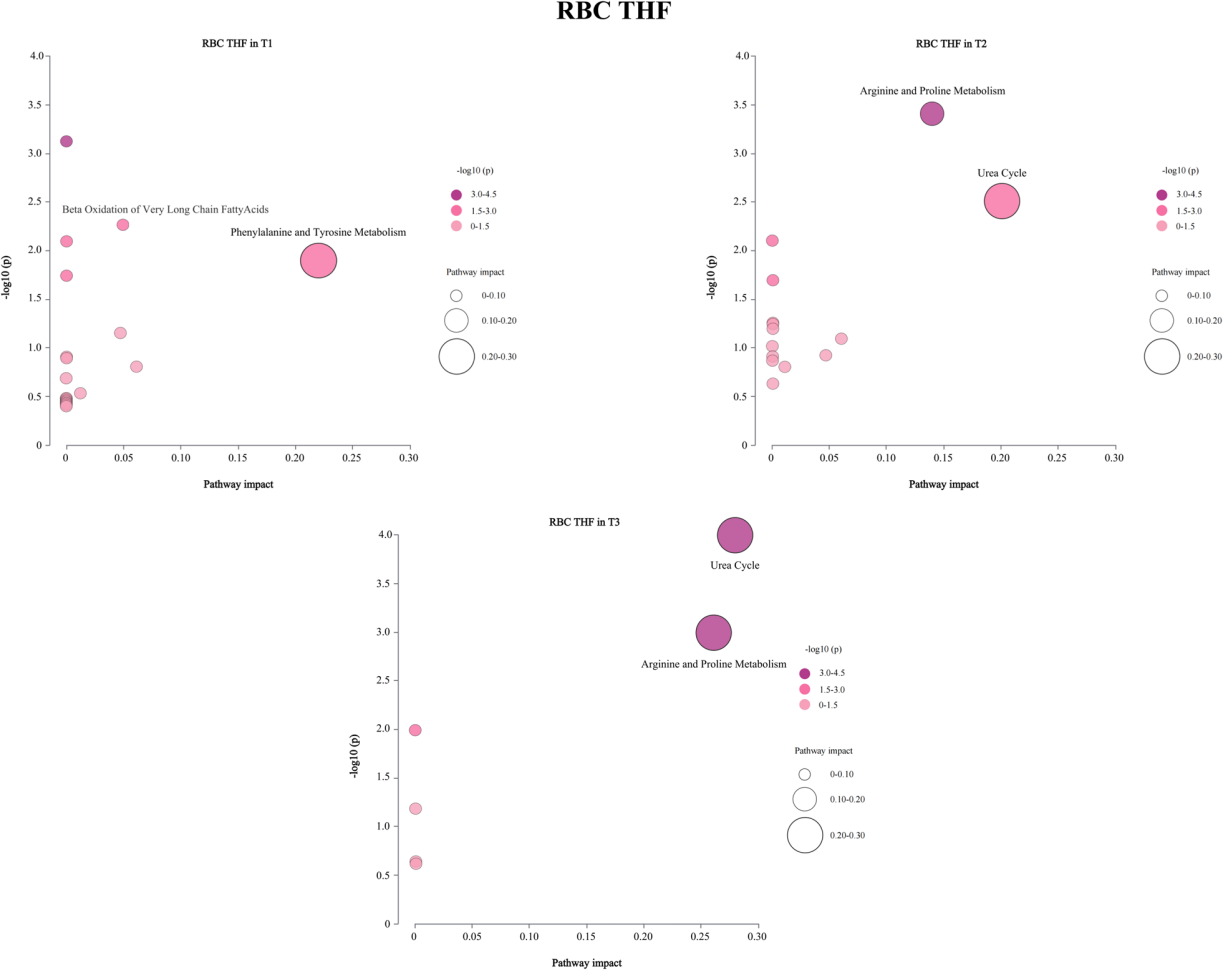
Associations between maternal RBC THF levels and neonatal metabolic pathways. The metabolic pathway name was labeled around the corresponding bubble, if the *p* value was less than 0.05 and the pathway impact was greater than 0

Total folate and 5-MTHF levels throughout pregnancy showed no associations with neonatal metabolites (all FDR > 0.05) (Supplementary Figures S1a and S1b). THF levels were associated with multiple neonatal metabolites, and the associations peaked in T1 and declined in T2 and T3. THF levels in T1 were positively related to phenylalanine (Phe) and tyrosine (Tyr) levels, involved in phenylalanine and tyrosine metabolism. And they were positively correlated with 7 ACs levels (C0, C4DC + C5-OH, C14, C14-OH, C14:1, C14:2, and C16-OH), and negatively with 9 ACs levels (C2, C3, C5DC + C6-OH, C6, C6DC, C12, C18, C18:1, and C18:2) (all FDR < 0.05), involved in fatty acids β-oxidation (*p* < 0.05). THF levels in T2 or T3 were positively associated with Arg and negatively with Cit, Gly, or ornithine (Orn) levels (all FDR < 0.05), engaged in urea cycle and arginine and proline metabolism (*p* < 0.05) (Figs. 4 and 5). It was worth noting that the impact of associations between THF levels and neonatal ACs were sex-specific and mainly manifested in T1. Specifically, the THF levels in T1 were negatively correlated with C2, C6DC, C18, C18:1 levels in male infants (all FDR < 0.05), while positively with C0, C14, C14-OH, C14:1, C14:2 levels in female infants (all FDR < 0.05). The correlations between THF levels in middle and late pregnancy and metabolites in male and female infants were not significant (Table S4). 5-CHO-THF displayed similar and weaker patterns compared to THF, associated with fatty acids β-oxidation only in T1 (Supplementary Figures S1c and S2a). These associations between UMFA levels and neonatal metabolic profiles differed from that of THF and 5-CHO-THF, related to urea cycle and arginine and proline metabolism only in T1 (Supplementary Figures S1d and S2b).

## Discussion

We revealed the dynamic influences of maternal folate metabolites on neonatal AAs and ACs, highlighting the crucial role of THF in early pregnancy at first. THF was significantly associated with neonatal phenylalanine and tyrosine metabolism and fatty acid β-oxidation pathways in early pregnancy, shifting to neonatal urea cycle and arginine and proline metabolism pathways in middle and late pregnancy. Compared to THF, 5-CHO-THF demonstrated similar but attenuated effects, significantly correlated with neonatal fatty acid β-oxidation pathways only in early pregnancy. UMFA exhibited different and weaker correlations, significantly related to neonatal urea cycle and arginine and proline metabolism pathways only in early pregnancy. Notably, neither 5-MTHF nor total folate showed significant correlations.

In our Beijing cohort study, the RBC total folate levels in early pregnancy [803.71 (543.81–1088.11) nmol/L] were generally aligned with values reported in Shanghai [788 (557–1094) nmol/L] [14] and Beijing (690.6 ± 258.3 nmol/L) [38], China. The RBC total levels in middle pregnancy (T2: 1137.24 [845.04, 1415.63] nmol/L) were slightly lower than those reported in Jiangsu (1478.83 [1124.60, 1865.71] nmol/L), China [39], potentially due to regional differences. Notably, our measurements were substantially lower than those reported in a Canadian study [mid-pregnancy: 2440 (2109, 2853) nmol/L; late pregnancy: 2929 (2522–3238) nmol/L] [40], which may be attributed to ethnic variations and differences in national fortification policies. Furthermore, previous studies indicated that the concentration of RBC total folate levels exhibited a rapid increase from early to middle pregnancy [14, 38, 39] followed by a more gradual rise through late pregnancy [40], which is generally consistent with the trend found in our research. This trend may reflect physiological adaptations to increasing fetal demands, where early pregnancy requires rapid folate pool expansion to support embryogenesis, while later stages prioritize sustained transfer to meet growing fetal requirements [41]. The agreement with other Chinese studies supports the reliability of our measurement method, while the observed variations with western populations underscore the importance of establishing population-specific reference ranges for accurate clinical interpretation.

Previous research reported that AAs and fatty acids in offspring exposed to maternal FA supplement were altered. Folate may be transported through the placenta, and it affects amino acid and lipid metabolism through one carbon metabolism and methionine cycle [19, 20] (Fig. 1). Our research further discovered that it was maternal THF levels that were significantly associated with a diverse range of neonatal AAs and ACs, particularly in early pregnancy. This may be due to its biological effect of supporting DNA synthesis and repair, enabling cell proliferation and differentiation [3], but fetal nucleotide synthesis gradually decelerates as gestation progresses [42]. It was worth noting that the impact of THF levels on neonatal ACs was sex-specific and mainly manifested in early pregnancy. Specifically, the THF levels in early pregnancy were positively correlated with C0, C14, C14-OH, C14:1, C14:2 levels in female infants, while negatively with negatively with C2, C6DC, C18, C18:1 levels in male infants. The positive correlations in female infants may be explained by the combined effect of elevated SAM under high folate availability and estrogen-mediated upregulation of PEMT activity, which may synergistically enhance phosphatidylcholine (PC) synthesis and thereby promote lipid metabolism [21, 22]. Furthermore, maternal THF levels were significantly correlated with neonatal phenylalanine and tyrosine metabolism and fatty acid β-xidation pathway in early pregnancy, whereas related to urea cycle and arginine and proline metabolism subsequently. This may align with evolving fetal demands, prioritizing neurodevelopment initially and nitrogen metabolism for protein synthesis later [43, 44]. These metabolic pathways are indirectly linked to the tricarboxylic acid (TCA) cycle and energy production, vital for disease progression [45].

In this study, elevated THF levels in early pregnancy were associated with increased Phe and Tyr levels in neonates, involved in the phenylalanine and tyrosine metabolism. Under the catalysis of phenylalanine hydroxylase (PAH), Phe is converted into Tyr, which is then transformed into levodopa by tyrosine hydroxylase (TH). These processes rely on the coenzyme tetrahydrobiopterin (BH4), whose regeneration is affected by folate metabolism [46]. Abnormal folate metabolism might impair the activity of key enzymes PAH and TH, leading to the accumulation of Phe and restricted conversion of Tyr. This imbalance was linked to neurological diseases and metabolic diseases, potentially mediated by hyperphenylalaninemia, phenylpyruvic acid production, and insufficient dopamine generation [47, 48].

ACs act as carriers of fatty acids into mitochondria and promote fatty acid β-oxidation. This research discovered that THF levels in early pregnancy impacted neonatal ACs profiles. High THF levels promoted C0 and long-chain ACs (C14-C16), possibly indicating enhanced fatty acid β-oxidation, whereas optimizing the short-chain ACs (C2-C6) clearance. The negative correlations with C18 ACs were likely due to the peroxisomal chain-shortening preprocessing [49]. These findings might imply folate's chain-length-dependent modulation of ACs, but specific mechanisms remained unclear. Fatty acid β-oxidation exerted dual effects, its enhancement supported tumorigenesis [50] but also suppressed cancers [51]. Similarly, its activation alleviated obesity [52] but also occurred in the early stages of obesity, accompanied by the upregulated carnitine palmitoyltransferase 1 A (Cpt1a), and the aberrant expression of TCA cycle and electron transport chain (ETC) related genes [53].

During middle and late pregnancy, THF levels were positively correlated with neonatal Arg levels and negatively with Cit, Orn, or Gly levels, involved in the urea cycle and arginine and proline metabolism. Urea cycle, as one of the main pathways of nitrogen metabolism, is closely related to arginine metabolism. Our results suggested that high folate levels might enhance urea cycle efficiency, accelerating Cit to Arg conversion and reducing Cit and Orn retention. A previous study found that FA supplementation was related to the urea cycle [54], whose disruptions contributed to disease pathogenesis. Urea cycle activation drove colorectal tumorigenesis [55], highly reliant on Arg availability [56]. And its disorder caused hyperammonemi, triggering neurological and psychiatric symptoms [57], and decreased cardiac function [58].

The associations of maternal 5-CHO-THF and UMFA levels with neonatal metabolites were also clarified in our study. 5-CHO-THF, a direct downstream derivative of THF, displayed similar but comparatively weaker effects than THF. UMFA, an inactive form of folate, may arise from excessive folate intake or folate metabolism disorder. While it might indicate adequate folate intake to some extent, its presence could pose potential harm by interfering with folate metabolism [59]. Our study showed UMFA levels were significantly connected with neonatal urea cycle and arginine and proline metabolism only in early pregnancy, but the effects disappeared in middle to late pregnancy, possibly due to UMFA conversion and redistribution caused by increased fetal nutrient demand and upregulated placental folate transporters [41]. Although prior animal studies suggested that maternal total folate intake influenced AAs and fatty acids levels in offspring [19, 20], we did not observe associations with RBC total folate or 5-MTHF levels in humans. This discrepancy may result from three factors: first, dietary intake does not completely translate to functional folate levels due to the biological complexity of folate metabolism; second, THF, 5-CHO-THF, and UMFA, which collectively represent a minor fraction of total folate, may exert disproportionate effects obscured by conventional “total folate” indicator; and third, existing animal models are limited in their ability to mimic human metabolic outcomes.

The developmental origins of health and disease hypothesis suggests that fetal programming is significantly influenced by the intrauterine environment. A previous study showed that maternal folate levels might influence metabolism in future generations through altered methylation patterns [9]. A prospective cohort study found that FA supplementation after the 12th week of pregnancy impacted DNA methylation in infants, potentially altering susceptibility to chronic diseases [60]. Rodent studies revealed that maternal FA supplementation significantly affected global and gene-specific methylation in offspring [61]. Excessive FA intake during pregnancy led to metabolic dysfunction in offspring, possibly mediated by the regulation of key metabolic enzymes [53, 62]. However, we observed that THF, crucial in nucleotide synthesis and methylation reactions, demonstrated a significant association, particularly in early pregnancy—a critical period of fetal nucleic acid synthesis [42], whereas 5-MTHF, pivotal in methylation processes, was not correlated with neonatal metabolism. Given that previous studies have primarily focused on FA supplementation and that this study only examined a portion of neonatal metabolites, we reasonably speculate that the intergenerational impacts of maternal folate levels partly operate through DNA synthesis, rather than DNA methylation. This perspective is supported by several studies. A population-based research discovered folate levels was not associated with neonatal DNA methylation [63]. A mouse experiment demonstrated that either maternal folate deficiency or excess resulted in disruptions in folate metabolism of the offspring, suggesting a diversion of the folate cycle from methylation to DNA synthesis [64].

## Strengths and limitations

Our study encompassed a large sample size of 4130 singleton pregnant women and their neonates. Rather than focusing on FA supplementation, we explored the associations between total folate and various folate metabolites in RBC and neonatal metabolites, which helped objectively determine the role of specific folate metabolites. Moreover, the prospective cohort design allowed us to evaluate the dynamic relationships between maternal folate metabolite levels at three stages of pregnancy and neonatal metabolites. Additionally, previous studies primarily relied on animal models to investigate the effects of maternal FA supplementation on offspring metabolism, our study explored the association with folate metabolites in a human cohort. However, this study has several limitations. First, the single-center design may restrict the generalizability of the results, necessitating further validation through multicenter studies with large sample sizes in the future. Second, we only detected two categories of neonatal metabolites, overlooking other potential differential metabolites. Finally, we only assessed maternal FA intake from supplements at enrollment, we did not evaluate the impact of total folate intake (e.g., dietary folate equivalents) from both diet and supplements on neonatal metabolites. Future studies should combine standardized dietary assessments (e.g., 24-h recalls or food frequency questionnaires) with all forms of folate intake from supplements to fully assess their effects.

## Conclusions

Although maternal total folate and 5-MTHF levels showed no significant association with neonatal metabolites, certain folate metabolites exhibited obvious trimester-specific associations with neonatal metabolic pathways. Maternal THF levels were significantly associated with neonatal phenylalanine and tyrosine metabolism and fatty acid β-oxidation pathways in early pregnancy, shifting to neonatal urea cycle and arginine and proline metabolism pathways in middle and late pregnancy. 5-CHO-THF and UMFA levels showed weaker effects. Additionally, the associations between maternal THF levels and neonatal ACs ​were​ sex-specific and mainly manifested​ in early pregnancy. These findings highlight THF may emerge as a critical regulator of fetal metabolic programming, suggesting that clinical monitoring of specific folate metabolites, beyond total folate, may be helpful for improving neonatal metabolic health.

## Supplementary Information

Additional file 1.

## Data Availability

The datasets used and analysed during the current study are available from the corresponding author (Guanghui Li, email: liguanghui@ccmu.edu.cn) on reasonable request.

## Abbreviations

FA: Folic acid
DHF: Dihydrofolate
THF: Tetrahydrofolate
5-CHO-THF: 5-Formyltetrahydrofolate
5-MTHF: 5-Methyltetrahydrofolate
UMFA: Unmetabolized folic acid
dUMP: Deoxyuridine monophosphate
dTMP: Deoxythymidine monophosphate
Hcy: Homocysteine
MTHFR: Methylenetetrahydrofolate reductase
MTRR: Methionine synthase reductase
NTDs: Neural tube defects
CHD: Congenital heart defect
RBC: Red blood cell
AAs: Amino acids
ACs: Acylcarnitines
BMI: Body mass index
AA: Arachidonic acid
D5D: Δ5-Desaturase
PE: Phosphatidylethanolamine
PC: Phosphatidylcholine
PEMT: Phosphatidylethanolamine N-methyltransferase
Met: Methionine
SAM: S-Adenosylmethionine
SAH: S-Adenosylhomocysteine
SHMT: Serine hydroxymethyltransferase
T1: 6-17 Gestational Weeks
T2: 20-26 Gestational Weeks
T3: 32-36 Gestational Weeks
LC‐MS/MS: Liquid chromatography-tandem mass spectrometry
UPLC: Ultra performance liquid chromatography
PCR: Polymerase chain reaction
HDP: Hypertensive disorders in pregnancy
GDM: Gestational diabetes mellitus
OGTT: Oral glucose tolerance test
LGA: Large for gestational age
SGA: Small for gestational age
LBW: Low birth weight
FDR: False discovery rate
SMPDB: Small Molecule Pathway Database
HMDB: Human Metabolome Database
Ala: Alanine
Gly: Glycine
Arg: Arginine
Cit: Citrulline
Phe: Phenylalanine
Tyr: Tyrosine
Orn: Ornithine
Leu + Ile + Pro-OH: Leucine + Isoleucine + Hydroxyproline
TCA: Tricarboxylic acid
PAH: Phenylalanine hydroxylase
TH: Tyrosine hydroxylase
BH4: Tetrahydrobiopterin
Cpt1a: Carnitine palmitoyltransferase 1A
ETC: Electron transport chain
