# Supplementary material for: Maternal red blood cell folate metabolites were dynamically associated with neonatal amino acids and acylcarnitines in heel blood: a prospective cohort study
Source: Nutr Metab (Lond). 2026 Jan 7;23:9. doi: 10.1186/s12986-025-01064-2 (PMC12809961; doi:10.1186/s12986-025-01064-2)
Supplement: Supplementary file 1 — Additional file 1. [file 12986_2025_1064_MOESM1_ESM.docx]

**Table S1** Maternal RBC folate metabolites levels at three stages of pregnancy

| Biochemical measurements | T1 (n=3222) | T2 (n=3287) | T3 (n=3211) | p  (T1 vs. T2) | p  (T2 vs. T3) |
| --- | --- | --- | --- | --- | --- |
| RBC total folate, nmol/L | 803.71 (543.81, 1088.11) | 1137.24 (845.04, 1415.63) | 1156.54 (820.83, 1453.10) | <0.001 | 0.226 |
| RBC 5‐MTHF, nmol/L | 765.43 (493.75, 1041.34) | 1081.42 (780.04, 1354.32) | 1093.42 (766.62, 1390.62) | <0.001 | 0.923 |
| RBC THF, nmol/L | 4.35 (1.70, 9.22) | 7.97 (3.74, 17.17) | 8.99 (4.37, 19.19) | <0.001 | <0.001 |
| RBC 5‐CHO‐THF, nmol/L | 13.82 (7.59, 26.06) | 20.55 (12.82, 35.57) | 21.16 (12.51, 38.79) | <0.001 | <0.001 |
| RBC UMFA, nmol/L | 7.55 (3.72, 16.88) | 6.30 (3.19, 14.39) | 6.07 (3.10, 12.40) | <0.001 | <0.001 |
| Measured weeks, gesttaional weeks | 9.43  (8.00-13.86) | 23.00  (20.71-24.86) | 32.29  (30.57-34.00) | - | - |

Paired t-tests with Bonferroni correction were used to conduct self-comparison of maternal folate metabolites from T1 to T2, and from T2 to T3, a corrected significance threshold of p < 0.025 (0.05/2) was applied to account for multiple comparisons. RBC: red blood cell, 5‐MTHF: 5‐methyltetrahydrofolate, THF: tetrahydrofolate, 5‐CHO‐THF: 5‐ formyltetrahydrofolate, UMFA: unmetabolised folic acid, T1: 6-17 gestational weeks, T2: 20-26 gestational weeks, T3: 32-36 gestational weeks.

**Table S2** Neonatal amino acids and acylcarnitines levels of the study participants

| Characteristics (μmol/L) | All (N=4130) | After log conversion |
| --- | --- | --- |
| ALA | 339.09 (280.25-404.89) | 2.51 (2.43-2.59) |
| ARG | 20.50 (14.21-29.09) | 1.28 (1.13-1.42) |
| CIT | 14.45 (12.12-17.61) | 1.15 (1.08-1.23) |
| GLY | 441.59 (355.23-545.47) | 2.68 (2.60-2.77) |
| LEU+Ile+Pro-OH | 150.17 (125.65-182.19) | 2.15 (2.08-2.22) |
| MET | 27.43 (23.56-31.77) | 1.44 (1.37-1.49) |
| ORN | 136.96 (111.27-169.70) | 2.12 (2.03-2.22) |
| PHE | 51.23 (44.90-58.35) | 1.71 (1.66-1.77) |
| PRO | 188.90 (160.13-225.28) | 2.26 (2.19-2.33) |
| TYR | 103.92 (84.60-128.21) | 2.01 (1.92-2.10) |
| VAL | 152.61 (125.96-183.42) | 2.17 (2.09-2.24) |
| C0 | 18.04 (14.34-23.25) | 1.24 (1.14-1.34) |
| C2 | 18.10 (13.79-23.51) | 1.32 (1.21-1.42) |
| C3 | 1.30 (0.99-1.71) | 0.16 (0.05-0.27) |
| C3DC+C4-OH | 0.09 (0.07-0.15) | -0.89 (-1.05-0.77) |
| C4 | 0.18 (0.15-0.22) | -0.72 (-0.80-0.64) |
| C4DC+C5-OH | 0.18 (0.15-0.22) | -0.72 (-0.80-0.64) |
| C5 | 0.10 (0.08-0.14) | -1.05 (-1.10-0.92) |
| C5:1 | 0.01 (0.01-0.01) | -2.00 (-2.00--2.00) |
| C5DC+C6-OH | 0.11 (0.09-0.14) | -0.92 (-1.00-0.82) |
| C6 | 0.04 (0.03-0.05) | -1.40 (-1.52-1.30) |
| C6DC | 0.14 (0.11-0.18) | -0.85 (-0.96-0.74) |
| C8 | 0.05 (0.04-0.07) | -1.22 (-1.30-1.10) |
| SA | 0.62 (0.51-0.75) | -0.21 (-0.29-0.12) |
| C10 | 0.07 (0.05-0.09) | -1.10 (-1.22-1.00) |
| C10:1 | 0.06 (0.05-0.08) | -1.15 (-1.22-1.05) |
| C10:2 | 0.01 (0.01-0.01) | -2.00 (-2.00--2.00) |
| C12 | 0.06 (0.05-0.09) | -1.10 (-1.22-0.96) |
| C12:1 | 0.06 (0.04-0.09) | -1.10 (-1.30-1.00) |
| C14 | 0.14 (0.10-0.18) | -0.80 (-0.92-0.72) |
| C14-OH | 0.01 (0.00-0.01) | -2.00 (-2.00-1.70) |
| C14:1 | 0.07 (0.05-0.10) | -1.05 (-1.15-0.92) |
| C14:2 | 0.02 (0.01-0.02) | -1.70 (-1.70-1.52) |
| C16 | 2.41 (1.45-3.37) | 0.48 (0.35-0.57) |
| C16-OH | 0.01 (0.01-0.02) | -1.70 (-2.00-1.70) |
| C16:1 | 0.13 (0.06-0.20) | -0.74 (-0.92-0.64) |
| C16:1-OH | 0.03 (0.02-0.04) | -1.52 (-1.52-1.40) |
| C18 | 0.67 (0.49-0.87) | -0.11 (-0.21-0.01) |
| C18-OH | 0.01 (0.01-0.01) | -2.00 (-2.00--2.00) |
| C18:1 | 1.25 (0.96-1.57) | 0.14 (0.05-0.24) |
| C18:1-OH | 0.01 (0.01-0.02) | -1.70 (-2.00-1.70) |
| C18:2 | 0.23 (0.17-0.31) | -0.68 (-0.82-0.55) |

**Table S3** The reference range of neonatal amino acids and acylcarnitines of the study participants

| Neonatal metabolites | Reference range (μmol/L) |
| --- | --- |
| ALA | 140.00-700.00 |
| ARG | 1.20-70.00 |
| CIT | 6.00-40.00 |
| GLY | 180.00-1000.00 |
| LEU+Ile+Pro-OH | 85.00-330.00 |
| MET | 10.00-55.00 |
| ORN | 40.00-400.00 |
| PHE | 25.00-110.00 |
| PRO | 80.00-400.00 |
| TYR | 40.00-400.00 |
| VAL | 60.00-300.00 |
| C0 | 8.60-60.00 |
| C2 | 5.00-50.00 |
| C3 | 0.40-5.50 |
| C3DC+C4-OH | 0.00-0.44 |
| C4 | 0.08-0.55 |
| C4DC+C5-OH | 0.10-0.70 |
| C5 | 0.02-0.45 |
| C5:1 | 0.00-0.03 |
| C5DC+C6-OH | 0.05-0.25 |
| C6 | 0.00-0.10 |
| C6DC | 0.00-0.35 |
| C8 | 0.00-0.30 |
| SA | 0.10-1.20 |
| C10 | 0.00-0.32 |
| C10:1 | 0.00-0.17 |
| C10:2 | 0.00-0.05 |
| C12 | 0.00-0.35 |
| C12:1 | 0.00-0.35 |
| C14 | 0.00-0.45 |
| C14-OH | 0.00-0.05 |
| C14:1 | 0.00-0.35 |
| C14:2 | 0.00-0.07 |
| C16 | 0.30-7.00 |
| C16-OH | 0.00-0.06 |
| C16:1 | 0.00-0.45 |
| C16:1-OH | 0.00-0.09 |
| C18 | 0.15-1.60 |
| C18-OH | 0.00-0.03 |
| C18:1 | 0.30-3.00 |
| C18:1-OH | 0.00-0.05 |
| C18:2 | 0.05-0.70 |

**Table S4** The associations between maternal THF levels and neonatal metabolites

**Table S4-1** The associations between maternal THF levels in early pregnancy and metabolites in male infants

| Neonatal metabolites | β (95% CI) | FDR |
| --- | --- | --- |
| ALA | -0.001 (-0.011, 0.008) | 0.973 |
| ARG | -0.008 (-0.025, 0.010) | 0.897 |
| CIT | 0.005 (-0.005, 0.016) | 0.897 |
| GLY | -0.023 (-0.034, -0.011) | <0.001 |
| LEU+Ile+Pro-OH | 0.011 (0.002, 0.021) | 0.258 |
| MET | 0.017 (0.009, 0.025) | <0.001 |
| ORN | -0.002 (-0.014, 0.010) | 0.973 |
| PHE | 0.011 (0.003, 0.018) | 0.172 |
| PRO | -0.003 (-0.012, 0.006) | 0.897 |
| TYR | 0.013 (0.002, 0.024) | 0.258 |
| VAL | 0.017 (0.007, 0.026) | 0.043 |
| C0 | 0.013 (0, 0.025) | 0.504 |
| C2 | -0.023 (-0.037, -0.010) | 0.043 |
| C3 | -0.022 (-0.037, -0.008) | 0.086 |
| C3DC+C4-OH | 0 (-0.018, 0.019) | 0.973 |
| C4 | -0.008 (-0.017, 0.002) | 0.697 |
| C4DC+C5-OH | 0.013 (0.003, 0.023) | 0.215 |
| C5 | -0.009 (-0.023, 0.005) | 0.897 |
| C5:1 | 0 (-0.003, 0.002) | 0.973 |
| C5DC+C6-OH | -0.018 (-0.029, -0.006) | 0.129 |
| C6 | -0.014 (-0.026, -0.002) | 0.387 |
| C6DC | -0.034 (-0.046, -0.021) | <0.001 |
| C8 | -0.012 (-0.026, 0.001) | 0.580 |
| SA | -0.006 (-0.016, 0.003) | 0.897 |
| C10 | -0.011 (-0.027, 0.005) | 0.721 |
| C10:1 | -0.005 (-0.017, 0.007) | 0.897 |
| C10:2 | -0.005 (-0.016, 0.006) | 0.897 |
| C12 | -0.022 (-0.039, -0.005) | 0.279 |
| C12:1 | -0.008 (-0.028, 0.013) | 0.897 |
| C14 | -0.003 (-0.018, 0.012) | 0.973 |
| C14-OH | 0.021 (0.007, 0.035) | 0.129 |
| C14:1 | 0.014 (-0.005, 0.032) | 0.721 |
| C14:2 | 0.019 (0.004, 0.034) | 0.258 |
| C16 | -0.025 (-0.046, -0.004) | 0.301 |
| C16-OH | 0.009 (-0.005, 0.024) | 0.897 |
| C16:1 | -0.036 (-0.062, -0.01) | 0.200 |
| C16:1-OH | -0.008 (-0.023, 0.006) | 0.897 |
| C18 | -0.028 (-0.044, -0.012) | 0.043 |
| C18-OH | -0.004 (-0.014, 0.007) | 0.897 |
| C18:1 | -0.028 (-0.042, -0.014) | <0.001 |
| C18:1-OH | 0 (-0.014, 0.013) | 0.973 |
| C18:2 | -0.014 (-0.029, 0.001) | 0.532 |

**Table S4-2** The associations between maternal THF levels in early pregnancy and metabolites in female infants

| Neonatal metabolites | β (95% CI) | FDR |
| --- | --- | --- |
| ALA | -0.006 (-0.016, 0.003) | 0.600 |
| ARG | -0.001 (-0.021, 0.020) | 0.995 |
| CIT | -0.002 (-0.013, 0.009) | 0.936 |
| GLY | -0.024 (-0.035, -0.013) | <0.001 |
| LEU+Ile+Pro-OH | 0.002 (-0.007, 0.012) | 0.847 |
| MET | 0.005 (-0.003, 0.014) | 0.600 |
| ORN | -0.010 (-0.022, 0.001) | 0.335 |
| PHE | 0.005 (-0.002, 0.012) | 0.600 |
| PRO | -0.010 (-0.019, -0.001) | 0.249 |
| TYR | 0.007 (-0.004, 0.018) | 0.600 |
| VAL | 0.014 (0.004, 0.024) | 0.108 |
| C0 | 0.024 (0.011, 0.037) | <0.001 |
| C2 | -0.005 (-0.018, 0.009) | 0.773 |
| C3 | -0.004 (-0.019, 0.012) | 0.847 |
| C3DC+C4-OH | 0.014 (-0.004, 0.032) | 0.519 |
| C4 | 0.006 (-0.004, 0.017) | 0.600 |
| C4DC+C5-OH | 0.010 (-0.001, 0.020) | 0.320 |
| C5 | -0.014 (-0.028, -0.001) | 0.293 |
| C5:1 | 0.002 (-0.001, 0.006) | 0.600 |
| C5DC+C6-OH | -0.010 (-0.022, 0.002) | 0.519 |
| C6 | -0.009 (-0.021, 0.004) | 0.600 |
| C6DC | -0.043 (-0.056, -0.03) | <0.001 |
| C8 | -0.008 (-0.022, 0.007) | 0.626 |
| SA | -0.001 (-0.01, 0.009) | 0.995 |
| C10 | -0.008 (-0.024, 0.008) | 0.731 |
| C10:1 | -0.002 (-0.014, 0.010) | 0.936 |
| C10:2 | 0 (-0.010, 0.010) | 0.995 |
| C12 | -0.007 (-0.025, 0.010) | 0.731 |
| C12:1 | 0.006 (-0.014, 0.026) | 0.805 |
| C14 | 0.029 (0.014, 0.045) | <0.001 |
| C14-OH | 0.026 (0.011, 0.041) | <0.001 |
| C14:1 | 0.033 (0.014, 0.051) | 0.043 |
| C14:2 | 0.036 (0.021, 0.051) | <0.001 |
| C16 | 0.004 (-0.018, 0.026) | 0.847 |
| C16-OH | 0.016 (0.002, 0.031) | 0.249 |
| C16:1 | 0.001 (-0.026, 0.027) | 0.995 |
| C16:1-OH | 0.012 (-0.002, 0.026) | 0.490 |
| C18 | -0.01 0(-0.026, 0.006) | 0.600 |
| C18-OH | 0.010 (-0.001, 0.021) | 0.320 |
| C18:1 | -0.012 (-0.026, 0.003) | 0.519 |
| C18:1-OH | 0.007 (-0.007, 0.021) | 0.731 |
| C18:2 | -0.012 (-0.028, 0.003) | 0.519 |

**Table S4-3** The associations between maternal THF levels in middle pregnancy and metabolites in male infants

| Neonatal metabolites | β (95% CI) | FDR |
| --- | --- | --- |
| ALA | -0.015 (-0.026, -0.003) | 0.105 |
| ARG | -0.001 (-0.024, 0.021) | 0.969 |
| CIT | -0.007 (-0.020, 0.005) | 0.679 |
| GLY | -0.019 (-0.033, -0.005) | 0.105 |
| LEU+Ile+Pro-OH | -0.008 (-0.019, 0.003) | 0.610 |
| MET | -0.003 (-0.013, 0.007) | 0.856 |
| ORN | -0.022 (-0.036, -0.008) | 0.105 |
| PHE | -0.007 (-0.016, 0.001) | 0.493 |
| PRO | -0.011 (-0.021, 0) | 0.371 |
| TYR | -0.012 (-0.026, 0.001) | 0.410 |
| VAL | -0.008 (-0.02, 0.003) | 0.610 |
| C0 | -0.004 (-0.019, 0.012) | 0.882 |
| C2 | 0.007 (-0.010, 0.023) | 0.756 |
| C3 | -0.002 (-0.020, 0.015) | 0.969 |
| C3DC+C4-OH | 0.004 (-0.019, 0.026) | 0.938 |
| C4 | 0.005 (-0.007, 0.016) | 0.756 |
| C4DC+C5-OH | -0.001 (-0.013, 0.011) | 0.969 |
| C5 | -0.011 (-0.028, 0.006) | 0.679 |
| C5:1 | 0 (-0.002, 0.003) | 0.938 |
| C5DC+C6-OH | -0.008 (-0.022, 0.006) | 0.679 |
| C6 | 0.014 (-0.001, 0.029) | 0.410 |
| C6DC | 0.014 (-0.001, 0.029) | 0.410 |
| C8 | 0.002 (-0.014, 0.019) | 0.938 |
| SA | -0.010 (-0.021, 0.001) | 0.410 |
| C10 | 0.007 (-0.012, 0.026) | 0.756 |
| C10:1 | -0.003 (-0.018, 0.012) | 0.882 |
| C10:2 | 0.001 (-0.013, 0.015) | 0.969 |
| C12 | 0.016 (-0.005, 0.037) | 0.610 |
| C12:1 | -0.010 (-0.034, 0.015) | 0.756 |
| C14 | 0.011 (-0.007, 0.030) | 0.679 |
| C14-OH | 0.006 (-0.010, 0.023) | 0.756 |
| C14:1 | 0.012 (-0.011, 0.035) | 0.679 |
| C14:2 | 0.015 (-0.003, 0.033) | 0.554 |
| C16 | -0.001 (-0.026, 0.025) | 0.969 |
| C16-OH | 0.005 (-0.013, 0.023) | 0.856 |
| C16:1 | 0.007 (-0.025, 0.038) | 0.882 |
| C16:1-OH | -0.011 (-0.028, 0.006) | 0.679 |
| C18 | -0.003 (-0.023, 0.016) | 0.938 |
| C18-OH | -0.006 (-0.019, 0.008) | 0.756 |
| C18:1 | -0.005 (-0.021, 0.012) | 0.856 |
| C18:1-OH | -0.004 (-0.020, 0.013) | 0.882 |
| C18:2 | -0.007 (-0.025, 0.012) | 0.756 |

**Table S4-4** The associations between maternal THF levels in middle pregnancy and metabolites in female infants

| Neonatal metabolites | β (95% CI) | FDR |
| --- | --- | --- |
| ALA | -0.009 (-0.019, 0.002) | 0.477 |
| ARG | 0.016 (-0.007, 0.04) | 0.562 |
| CIT | -0.016 (-0.028, -0.004) | 0.172 |
| GLY | -0.019 (-0.032, -0.006) | 0.172 |
| LEU+Ile+Pro-OH | 0.004 (-0.007, 0.014) | 0.882 |
| MET | 0.007 (-0.002, 0.017) | 0.477 |
| ORN | -0.013 (-0.026, 0) | 0.336 |
| PHE | -0.008 (-0.016, 0) | 0.336 |
| PRO | 0.006 (-0.005, 0.016) | 0.706 |
| TYR | 0.002 (-0.010, 0.015) | 0.933 |
| VAL | 0.003 (-0.008, 0.014) | 0.882 |
| C0 | -0.015 (-0.029, 0) | 0.336 |
| C2 | 0.004 (-0.012, 0.019) | 0.882 |
| C3 | -0.002 (-0.019, 0.015) | 0.975 |
| C3DC+C4-OH | -0.009 (-0.030, 0.011) | 0.771 |
| C4 | 0 (-0.011, 0.012) | 0.975 |
| C4DC+C5-OH | -0.002 (-0.014, 0.009) | 0.933 |
| C5 | -0.001 (-0.017, 0.015) | 0.975 |
| C5:1 | 0 (-0.004, 0.003) | 0.975 |
| C5DC+C6-OH | -0.022 (-0.036, -0.009) | 0.043 |
| C6 | -0.003 (-0.017, 0.011) | 0.933 |
| C6DC | 0.010 (-0.004, 0.025) | 0.562 |
| C8 | -0.015 (-0.031, 0) | 0.366 |
| SA | -0.013 (-0.024, -0.003) | 0.258 |
| C10 | -0.010 (-0.028, 0.008) | 0.706 |
| C10:1 | -0.016 (-0.030, -0.002) | 0.322 |
| C10:2 | -0.003 (-0.014, 0.009) | 0.882 |
| C12 | 0.009 (-0.011, 0.029) | 0.771 |
| C12:1 | -0.021 (-0.044, 0.002) | 0.525 |
| C14 | 0.009 (-0.009, 0.026) | 0.771 |
| C14-OH | -0.001 (-0.017, 0.015) | 0.975 |
| C14:1 | 0.002 (-0.019, 0.023) | 0.975 |
| C14:2 | 0.007 (-0.010, 0.024) | 0.798 |
| C16 | -0.007 (-0.031, 0.017) | 0.882 |
| C16-OH | 0.001 (-0.015, 0.018) | 0.975 |
| C16:1 | 0 (-0.029, 0.030) | 0.975 |
| C16:1-OH | -0.009 (-0.024, 0.007) | 0.706 |
| C18 | -0.011 (-0.029, 0.007) | 0.706 |
| C18-OH | 0.001 (-0.012, 0.013) | 0.975 |
| C18:1 | -0.006 (-0.022, 0.010) | 0.798 |
| C18:1-OH | -0.014 (-0.030, 0.002) | 0.525 |
| C18:2 | -0.013 (-0.030, 0.005) | 0.525 |

**Table S4-5** The associations between maternal THF levels in late pregnancy and metabolites in male infants

| Neonatal metabolites | β (95% CI) | FDR |
| --- | --- | --- |
| ALA | 0 (-0.011, 0.010) | 0.992 |
| ARG | 0.027 (0.007, 0.047) | 0.147 |
| CIT | -0.008 (-0.019, 0.003) | 0.578 |
| GLY | 0.002 (-0.010, 0.014) | 0.948 |
| LEU+Ile+Pro-OH | 0.014 (0.004, 0.024) | 0.147 |
| MET | 0.014 (0.005, 0.023) | 0.084 |
| ORN | -0.008 (-0.020, 0.004) | 0.578 |
| PHE | 0.002 (-0.006, 0.009) | 0.948 |
| PRO | 0.010 (0, 0.020) | 0.420 |
| TYR | 0.006 (-0.006, 0.017) | 0.772 |
| VAL | 0.007 (-0.003, 0.017) | 0.578 |
| C0 | -0.003 (-0.017, 0.011) | 0.948 |
| C2 | 0.020 (0.005, 0.035) | 0.189 |
| C3 | -0.001 (-0.016, 0.015) | 0.992 |
| C3DC+C4-OH | 0.003 (-0.017, 0.024) | 0.948 |
| C4 | 0.014 (0.003, 0.024) | 0.189 |
| C4DC+C5-OH | 0.006 (-0.005, 0.016) | 0.772 |
| C5 | 0.004 (-0.011, 0.019) | 0.948 |
| C5:1 | -0.001 (-0.003, 0.002) | 0.948 |
| C5DC+C6-OH | -0.008 (-0.021, 0.005) | 0.578 |
| C6 | 0.011 (-0.002, 0.025) | 0.420 |
| C6DC | 0.018 (0.004, 0.031) | 0.189 |
| C8 | 0 (-0.015, 0.015) | 0.992 |
| SA | -0.018 (-0.028, -0.007) | 0.084 |
| C10 | 0.002 (-0.015, 0.020) | 0.992 |
| C10:1 | -0.010 (-0.023, 0.003) | 0.578 |
| C10:2 | -0.005 (-0.017, 0.007) | 0.815 |
| C12 | 0.020 (0.002, 0.039) | 0.420 |
| C12:1 | 0.001 (-0.021, 0.023) | 0.992 |
| C14 | 0.021 (0.004, 0.038) | 0.273 |
| C14-OH | -0.004 (-0.018, 0.011) | 0.948 |
| C14:1 | 0.014 (-0.006, 0.035) | 0.578 |
| C14:2 | 0.008 (-0.009, 0.024) | 0.772 |
| C16 | 0.002 (-0.020, 0.025) | 0.992 |
| C16-OH | -0.001 (-0.017, 0.015) | 0.992 |
| C16:1 | 0.015 (-0.013, 0.043) | 0.772 |
| C16:1-OH | -0.006 (-0.022, 0.009) | 0.815 |
| C18 | 0.009 (-0.008, 0.026) | 0.772 |
| C18-OH | 0.002 (-0.010, 0.014) | 0.948 |
| C18:1 | 0.010 (-0.005, 0.025) | 0.578 |
| C18:1-OH | 0.004 (-0.011, 0.019) | 0.948 |
| C18:2 | 0.007 (-0.009, 0.024) | 0.772 |

**Table S4-6** The associations between maternal THF levels in late pregnancy and metabolites in female infants

| Neonatal metabolites | β (95% CI) | FDR |
| --- | --- | --- |
| ALA | -0.008 (-0.018, 0.003) | 0.588 |
| ARG | 0.030 (0.007, 0.054) | 0.220 |
| CIT | -0.014 (-0.026, -0.002) | 0.280 |
| GLY | -0.009 (-0.021, 0.004) | 0.588 |
| LEU+Ile+Pro-OH | 0.011 (0.001, 0.022) | 0.429 |
| MET | 0.018 (0.008, 0.027) | <0.001 |
| ORN | -0.016 (-0.029, -0.004) | 0.231 |
| PHE | 0.002 (-0.006, 0.009) | 0.909 |
| PRO | 0.005 (-0.005, 0.015) | 0.767 |
| TYR | 0.006 (-0.007, 0.018) | 0.767 |
| VAL | 0.003 (-0.008, 0.014) | 0.880 |
| C0 | -0.011 (-0.025, 0.003) | 0.546 |
| C2 | 0 (-0.015, 0.016) | 0.955 |
| C3 | 0.009 (-0.008, 0.026) | 0.660 |
| C3DC+C4-OH | -0.019 (-0.040, 0.001) | 0.441 |
| C4 | 0.010 (-0.002, 0.022) | 0.493 |
| C4DC+C5-OH | 0.002 (-0.009, 0.013) | 0.909 |
| C5 | 0.020 (0.005, 0.035) | 0.220 |
| C5:1 | 0.002 (-0.002, 0.005) | 0.726 |
| C5DC+C6-OH | -0.012 (-0.025, 0.001) | 0.456 |
| C6 | 0.006 (-0.008, 0.02) | 0.767 |
| C6DC | 0.025 (0.011, 0.04) | 0.042 |
| C8 | -0.024 (-0.040, -0.008) | 0.126 |
| SA | -0.008 (-0.019, 0.002) | 0.546 |
| C10 | -0.012 (-0.030, 0.006) | 0.588 |
| C10:1 | -0.015 (-0.029, -0.002) | 0.420 |
| C10:2 | -0.012 (-0.024, -0.001) | 0.429 |
| C12 | 0.005 (-0.014, 0.025) | 0.880 |
| C12:1 | -0.010 (-0.032, 0.013) | 0.767 |
| C14 | -0.001 (-0.018, 0.017) | 0.955 |
| C14-OH | -0.010 (-0.025, 0.004) | 0.588 |
| C14:1 | 0.009 (-0.012, 0.030) | 0.767 |
| C14:2 | 0.009 (-0.008, 0.025) | 0.726 |
| C16 | -0.016 (-0.040, 0.008) | 0.588 |
| C16-OH | -0.011 (-0.028, 0.005) | 0.588 |
| C16:1 | -0.004 (-0.033, 0.025) | 0.909 |
| C16:1-OH | -0.001 (-0.017, 0.014) | 0.955 |
| C18 | -0.007 (-0.025, 0.010) | 0.767 |
| C18-OH | 0.001 (-0.011, 0.013) | 0.955 |
| C18:1 | -0.008 (-0.023, 0.008) | 0.726 |
| C18:1-OH | -0.010 (-0.025, 0.006) | 0.616 |
| C18:2 | -0.011 (-0.028, 0.007) | 0.616 |

1. **RBC total folate**

**
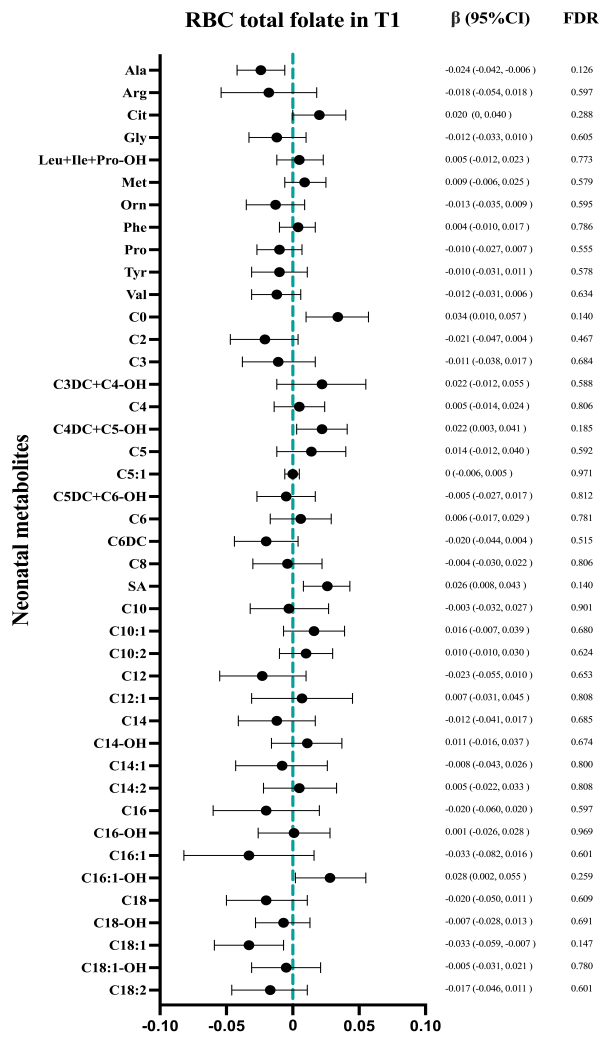

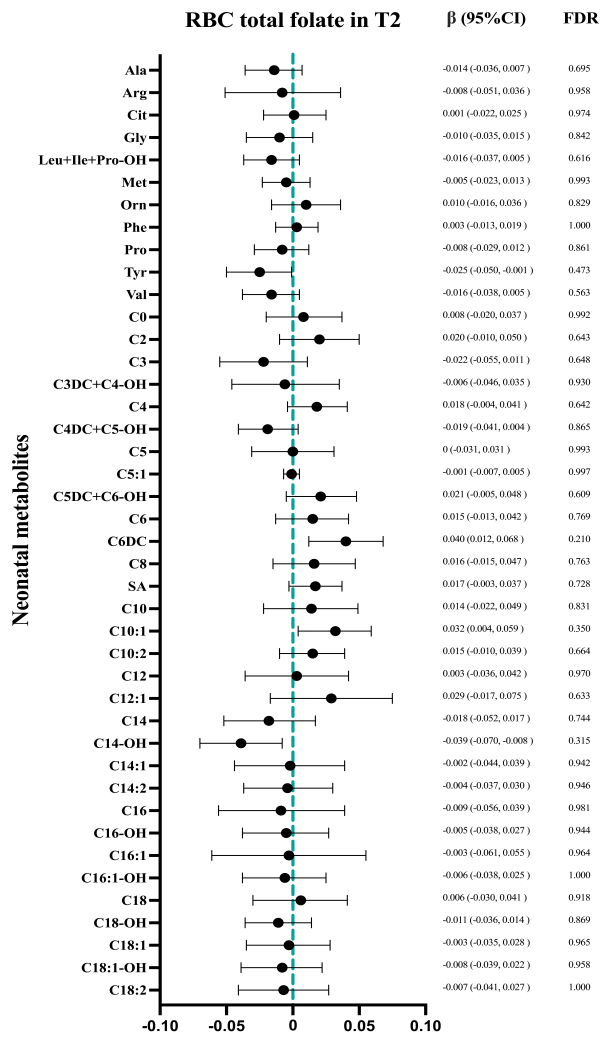

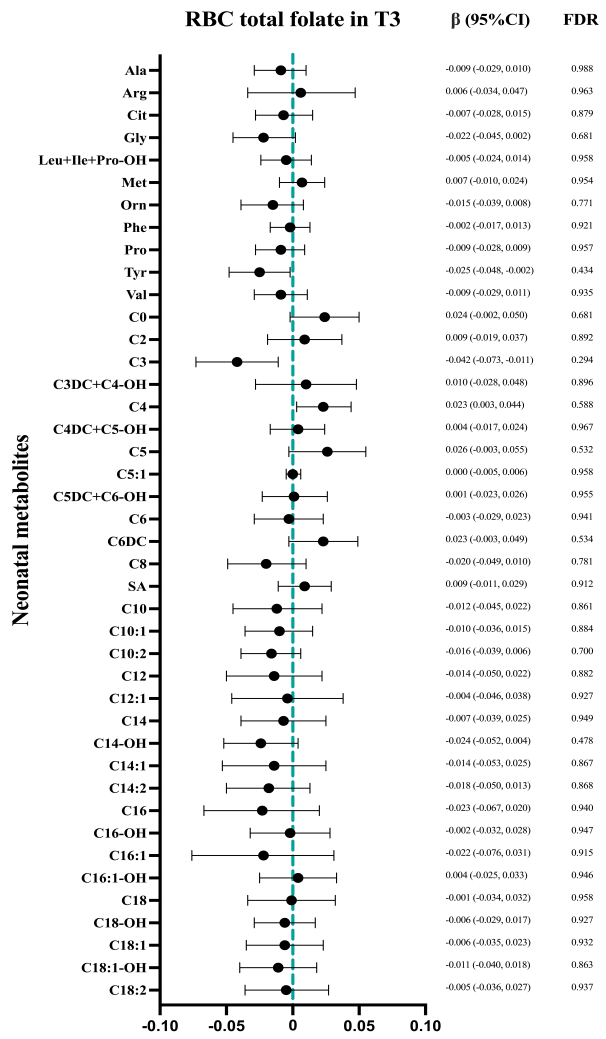
**

1. **RBC 5-MTHF**

**
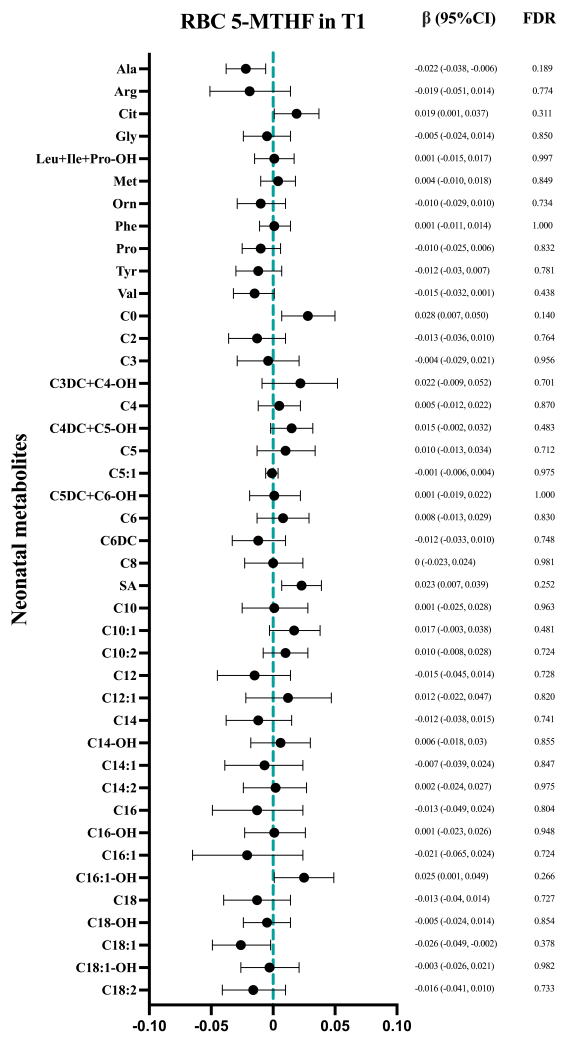

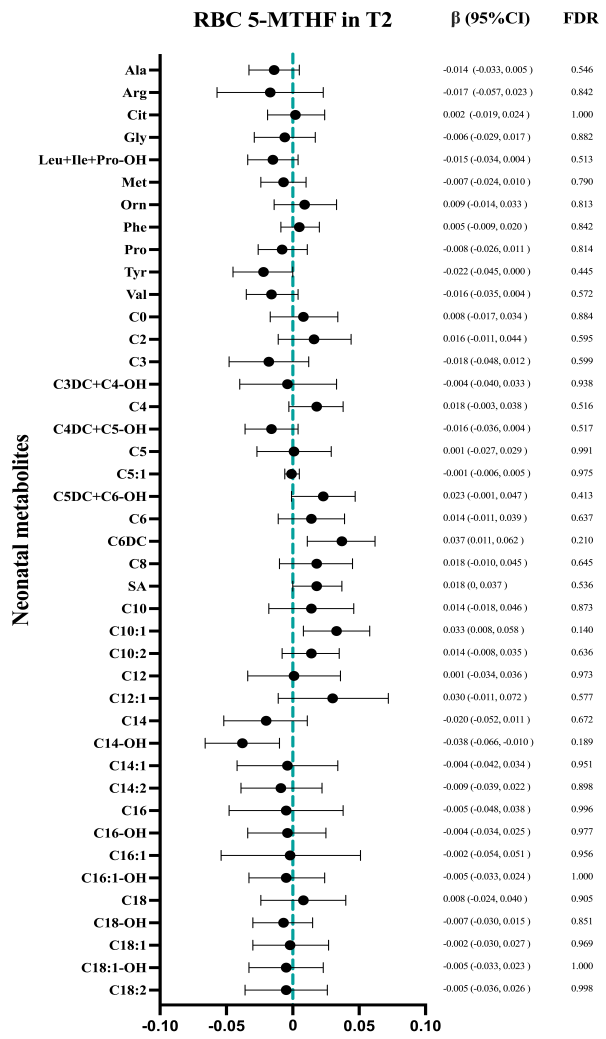
**

**
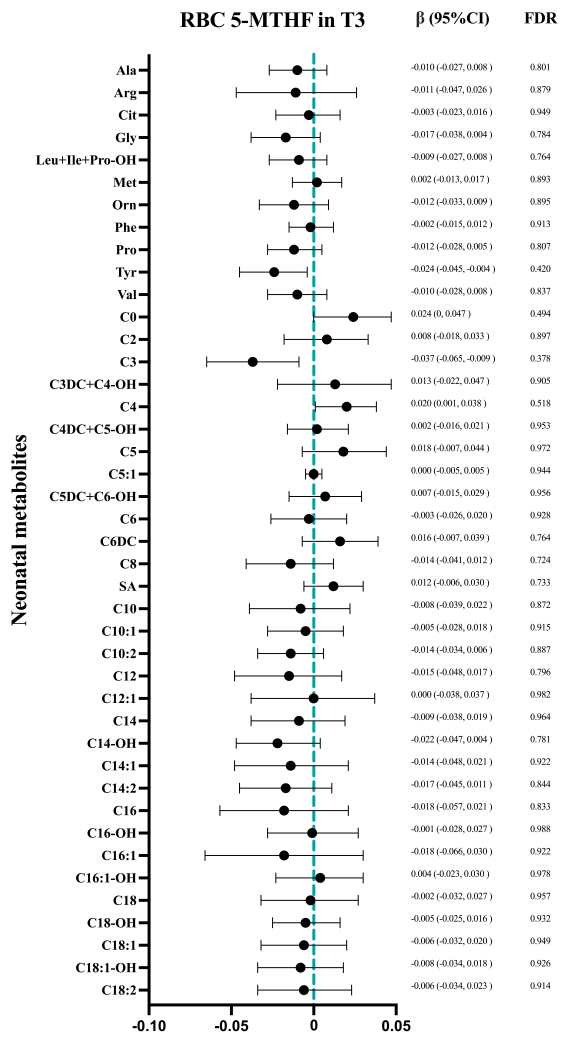
**

1. **RBC 5-CHO-THF**

**
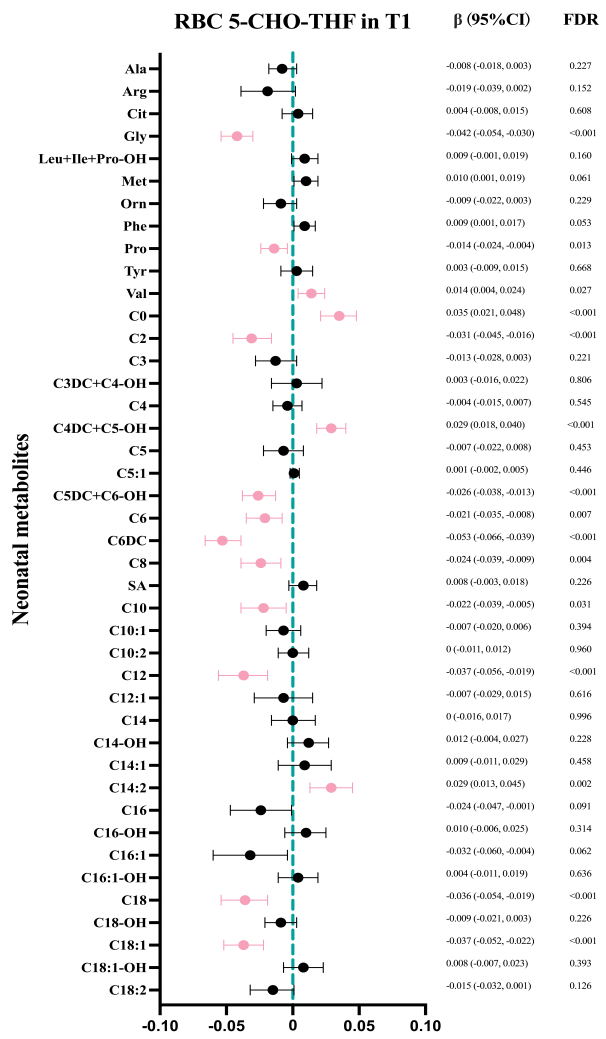

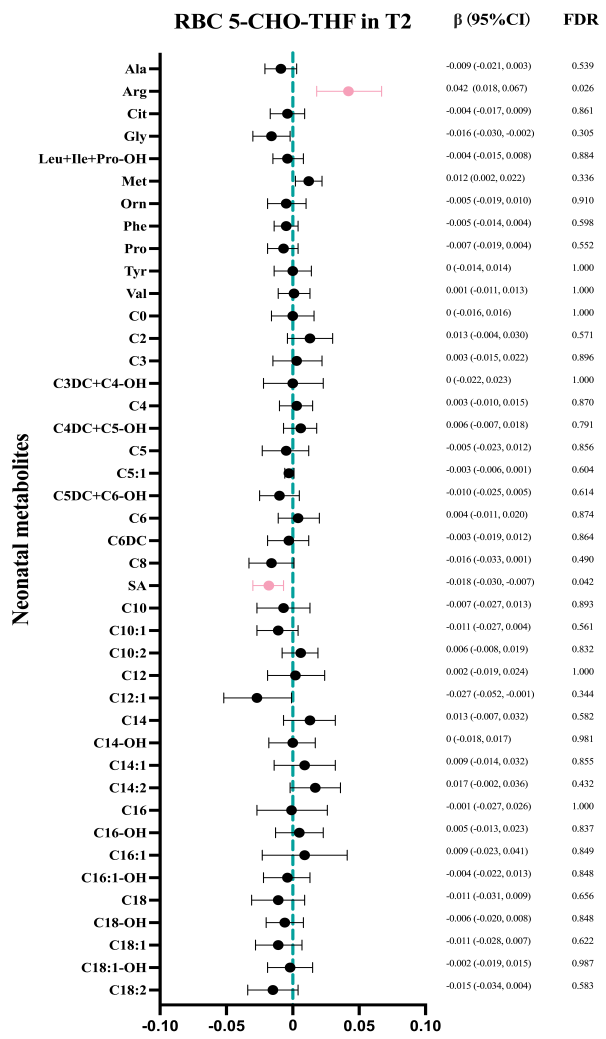

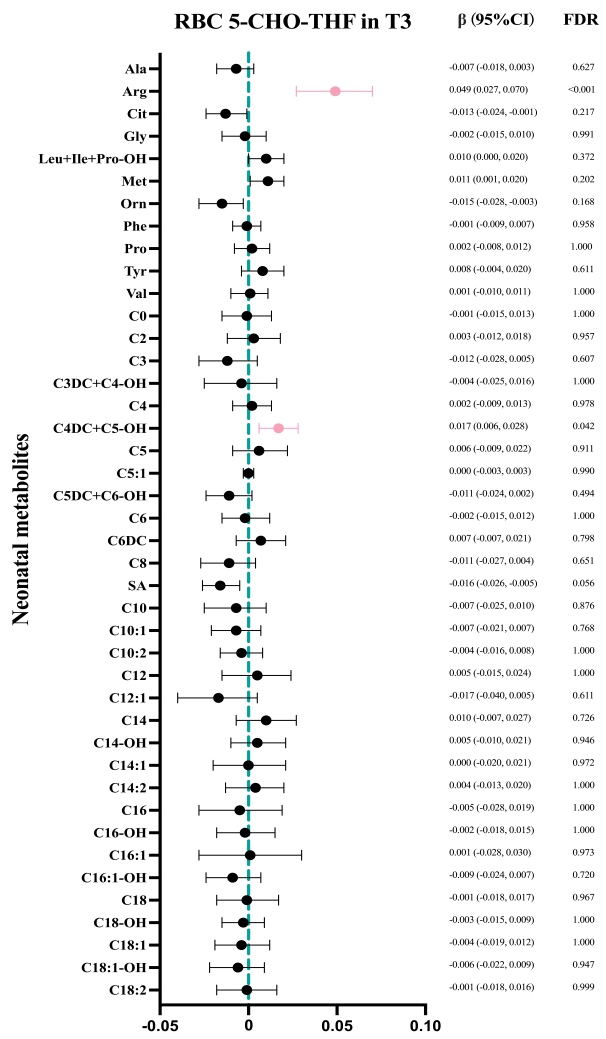
**

1. **RBC UMFA**


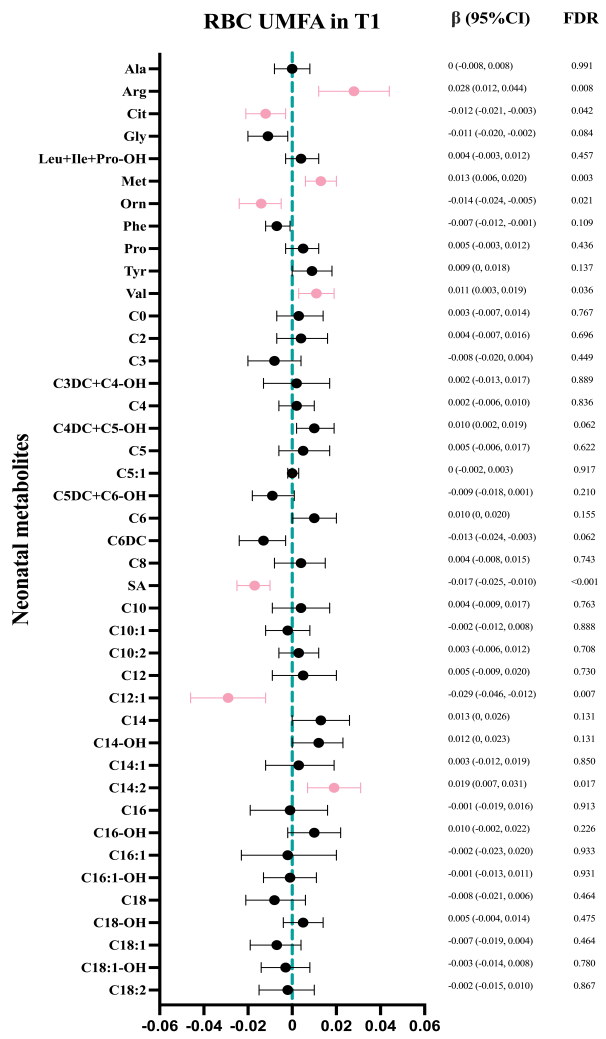

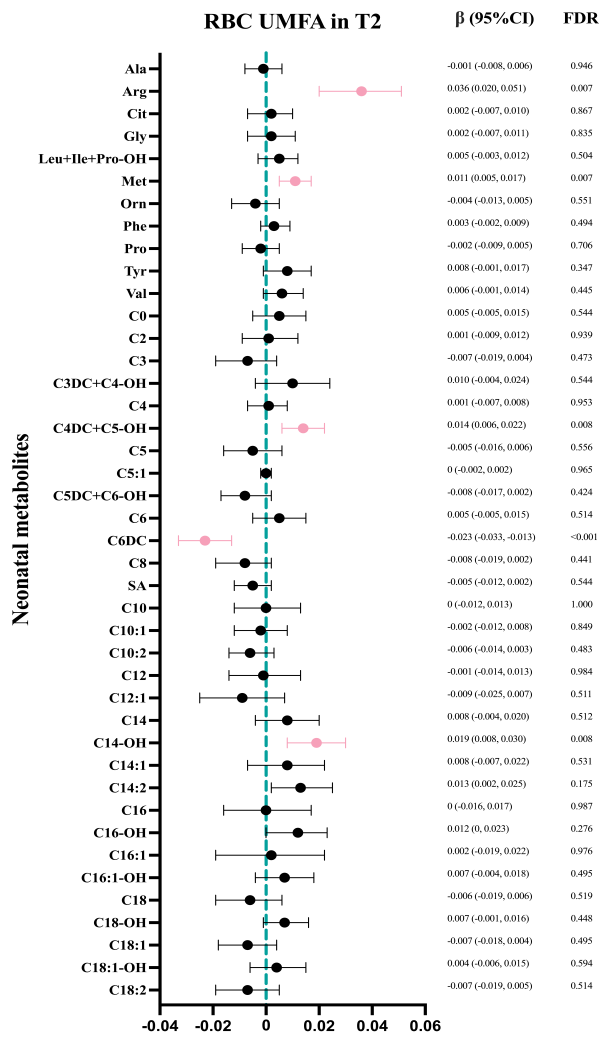

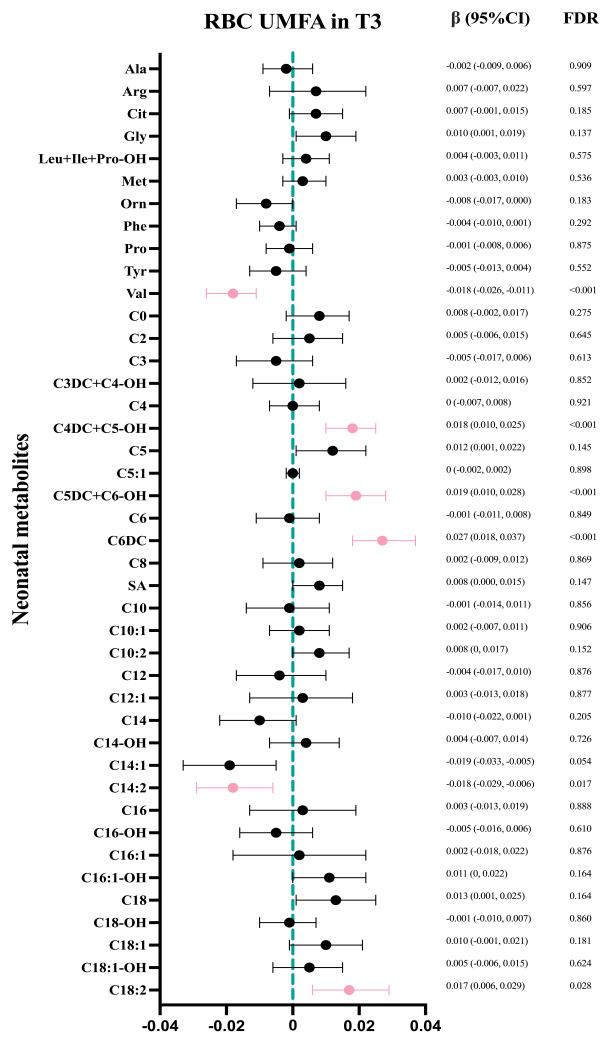


**Figure S1** Associations between maternal folate metabolites levels (a: RBC total folate; b: RBC 5-MTHF; c: RBC 5-CHO-THF; d: RBC UMFA ) and neonatal metabolites. Maternal age, pre-pregnancy BMI, parity, HDP, GDM, and neonatal gender were adjusted in linear regression analysis. The FDR values were calculated to account for multiple comparisons using the Benjamini-Hochberg method.
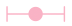
FDR<0.05.

**
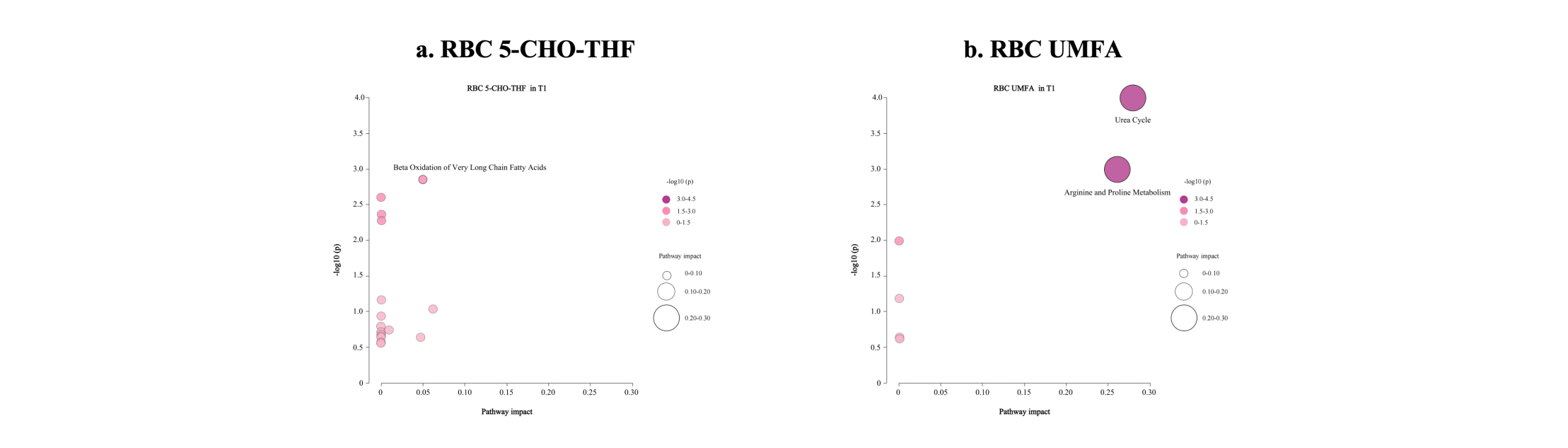
**

**Figure S2** Associations between maternal folate metabolites levels (a: RBC 5-CHO-THF; b: RBC UMFA) and neonatal metabolic pathways. The metabolic pathway name was labeled around the corresponding bubble, if the p value was less than 0.05 and the pathway impact was greater than 0. The metabolic pathways related to maternal 5-CHO-THF and UMFA were showed only in T1, because no significant changes in metabolic pathways existed in T2 and T3.
